# An updated systematic review of the impact of volume of surgery and specialization in Norwood procedure

**DOI:** 10.1186/s12887-026-07179-6

**Published:** 2026-06-24

**Authors:** Alexander Pachanov, Zhentian Zhang, Silvan Munschek, Boulos Asfour, Tim Mathes, Dawid Pieper

**Affiliations:** 1 Faculty of Health Sciences Brandenburg, Institute for Health Services and Health System Research, Brandenburg Medical School (Theodor Fontane), Seebad 82/83, Rüdersdorf bei Berlin, 15562 Germany; 2 Center for Health Services Research, Brandenburg Medical School (Theodor Fontane), Rüdersdorf, Germany; 3 Department of Medical Statistics, University Medical Center Göttingen, Gottingen, Germany; 4 The German Paediatric Heart Centre, University Hospital Bonn, Bonn, Germany

**Keywords:** HLHS, Hypoplastic left heart syndrome, Norwood procedure, Congenital heart surgery, Systematic review

## Abstract

**Background:**

Volume-outcome relationship proposed to exist for high-risk, low-volume procedures, such as the Norwood procedure. A systematic review published in 2014 examined impacts of hospital and surgeon volume, specialization, regionalization and teaching status on the patient-related outcomes of the Norwood procedure. The aim of this systematic review was to update the 2014 work by synthesizing current evidence alongside the original review.

**Methods:**

We searched PubMed, Embase (Elsevier), and the Cochrane Library for peer-reviewed comparative studies published from 1 March 2013 to 30 December 2024 (date of last search) to update the original review covering database inception to March 2013. Citation chasing of relevant reports was performed on 20 March 2025. Mortality-related outcomes were defined as primary and all others as secondary. In studies on the volume-outcome relationship with categorical volume definitions, effect estimates were compared between the highest and lowest categories, as defined in each study. Risk of bias and certainty of evidence were assessed using ROBINS-E and GRADE, respectively. Data were presented in tables and synthesized narratively.

**Results:**

Eight additional studies reported in 13 publications were identified, resulting in a total of 18 studies (24 reports). Of these, 15 studies (20 reports), comprising 47 analyses, were included in the final synthesis. The reports were published between 2002 and 2025 and predominantly relied on routinely collected data from North America. Irrespective of statistical significance, 14 of 15 short-term and 4 of 5 long-term hospital-volume analyses, and 3 of 5 short-term and 3 of 3 long-term surgeon-volume analyses of mortality-related outcomes favored higher volume. Among 17 secondary outcome analyses, 14 favored higher hospital or surgeon volume. Evidence on hospital teaching status was limited to two older studies, which reported lower mortality in teaching hospitals. Overall, the certainty of evidence was rated as very low, reflecting heterogeneity in exposure definitions, reliance on routinely collected data, and limited use of analytical approaches that support causal interpretation.

**Conclusions:**

Although most analyses favored higher hospital or surgeon volume and teaching hospital status, the very low certainty of evidence limits its ability to inform clinical practice or policy, underscoring the need for stronger methodological approaches in future research.

**Registration:**

PROSPERO CRD42022385160.

**Supplementary Information:**

The online version contains supplementary material available at 10.1186/s12887-026-07179-6.

## Background

### Rationale

Hypoplastic left heart syndrome (HLHS) is a group of severe congenital heart defects characterized by underdevelopment of the left-sided heart structures [1, 2]. Without surgical intervention, the condition is uniformly fatal in the neonatal period [3]. A three-stage surgical palliation is the standard of care for patients with HLHS and other functionally similar forms of univentricular heart lesions [4]. The Norwood procedure, which performed shortly after birth, is the most common stage I palliation procedure [3]. It is followed by the bidirectional Glenn or hemi-Fontan procedure at 3–6 months of age (stage II), and the Fontan completion at 2–4 years (stage III) [2].

Despite advances in congenital heart surgery (CHS) and perioperative care, operative and interstage mortality after the Norwood procedure remain substantial, reaching up to 30% [5–8], and the majority of patients experience postoperative complications [7, 9].

These high burdens have prompted investigations into system- and provider-level factors that may influence Norwood outcomes. Across surgical specialties, numerous studies have shown an association between procedural volume and patient outcomes [10–12], often explained by the “practice-makes-perfect” hypothesis. According to this hypothesis, repeated exposure to complex procedures allows surgeons and their teams to refine operative skills, optimize perioperative protocols, and strengthen multidisciplinary collaboration [13, 14].

If a volume-outcome relationship exists in the Norwood procedure, strategies such as regionalizing care, setting minimum volume thresholds, or designating specialized pediatric cardiac centers could help improve outcomes of surgical care for HLHS. Some countries are moving in this direction. For example, several European countries and Canada have already implemented centralization strategies in CHS, while others continue to debate whether the benefits of concentrating expertise and resources outweigh potential concerns about access and equity [15].

This debate underscores the importance of understanding whether such strategies truly drive better outcomes. A 2014 systematic review (SR) was the first to comprehensively assess the relationship between institutional volume, surgeon volume, regionalization, hospital teaching status and patient outcomes following the Norwood procedure [16]. The review included ten studies, most of which were observational using U.S.-based registry or administrative data. The results suggested that higher hospital volume was associated with lower mortality. Teaching hospitals also appeared to have better outcomes, although findings regarding surgeon volume were inconsistent.

Since 2014, additional studies may have been conducted that further explore these relationships, potentially incorporating bigger patient cohorts, applying stronger methodologies, or focusing on outcomes not addressed in the original review. Therefore, an updated SR can help determine whether more recent studies provide greater clarity on the volume-outcome relationship or the impact of hospital type on patient outcomes following the Norwood procedure.

### Objectives

With this SR, we aimed to update the 2014 review by synthesizing evidence from comparative studies of any design on the relationship between institutional or surgeon volume, regionalization, teaching hospital status, and patient outcomes among infants undergoing the Norwood procedure.

## Methods

### Protocol and registration

This systematic review is registered in the PROSPERO database (CRD42022385160) and reported in accordance with the Preferred Reporting Items for Systematic Reviews and Meta-Analyses 2020 (PRISMA-2020) [17].

### Eligibility criteria

We intended to include studies evaluating outcomes across hospital or surgeon procedural volume levels, hospital specialization or teaching status, and regionalization of care that met the following criteria:

- Population: Infants with HLHS or morphologically similar single ventricle conditions undergoing the Norwood procedure.
- Outcomes: mortality/survival and other patient-related/patient-reported postoperative outcomes or complications.
- Study design: Comparative observational or interventional studies of any design.

We focused primarily on volume-outcome studies that used Norwood volume as the exposure of interest. However, we also considered studies that examined Norwood-specific outcomes in relation to overall pediatric cardiac surgical volume, given its potential correlation with Norwood volume [18].

Exclusion criteria encompassed studies addressing other patient populations, surgical procedures, provider characteristics or non-comparative designs. Furthermore, we excluded editorials, reviews and conference abstracts.

### Information sources and search strategy

We searched PubMed, Embase (Elsevier) and Cochrane Library (all databases) from March 2013 (the last search conducted for the original SR). The initial searches were performed on 17 January 2023 and updated on 30 December 2024. On 20 March 2025, we performed forward and backward citation tracking using the Citationchaser Shiny app [19].

The search strategy was based on that used in the original SR. The full search strategies for all databases are presented in Supplementary file 1.

### Selection process

Study selection was performed independently by two reviewers at each stage using the Rayyan web application [20]. Discrepancies at any stage were resolved through discussion or, when necessary, by involving a third reviewer. Reasons for exclusion at the full-text screening stage were documented.

### Data collection process

A standardized data extraction form was developed based on the one used in the original SR. Due to resource constraints, data were extracted by one reviewer and checked for accuracy by a second. Discrepancies were resolved through discussion or, when necessary, by consultation with a third reviewer.

### Data items

The following data were extracted from each included study:

- Study identification (first author, year, country).
- Study data sources and data collection period.
- Study type, e.g. prospective vs. retrospective.
- Patient, hospital, surgeon characteristics.
- Definitions and categorizations of exposure (e.g., volume thresholds).
- Outcomes and effect estimates.
- Methods of statistical adjustment.

### Risk of bias assessment

Risk of bias in non-randomized studies was assessed independently by two reviewers, using the Risk Of Bias In Non-randomized Studies of Exposures (ROBINS-E) tool [21]. We considered studies to have sufficiently controlled for confounding if the authors adjusted for a relevant set of patient characteristics or risk factors [22], such as age at operation, birth weight, prematurity, sex, ethnicity, preoperative clinical status (e.g., shock, ventilatory support), the presence of additional cardiac malformations or non-cardiac comorbidities. If randomized controlled trials were included, we planned to use the Cochrane Risk of Bias 2 (RoB2) tool [23]. The 2014 SR’s risk of bias assessments were updated to these tools for consistency. Disagreements were resolved by discussion or involving a third reviewer. Risk of bias assessments were performed at the outcome level.

### Effect measures

When multiple effect estimates were reported in the original studies, we prioritized adjusted estimates from multivariable models. If multiple adjusted models were available, we included results from all relevant models. For dichotomous outcomes, effect measures included odds ratios (ORs) or hazard ratios (HRs), with 95% confidence intervals. For continuous outcomes, we used mean differences or standardized mean differences. If no adjusted estimates were reported, we extracted and reported crude rates, correlation coefficients or unadjusted risk estimates. In studies on the volume-outcome relationship with categorical volume definitions, effect estimates were compared between the highest and lowest categories, as defined in each study.

### Synthesis methods

We analyzed and summarized the included studies narratively, grouping them by exposure of interest (hospital or surgeon volume, hospital type) and the outcomes examined, as well as counted the number of analyses according to direction of effect and statistical significance. Our primary outcome of interest was mortality or survival (categorized into short-term and long-term outcomes), while secondary outcomes included other postoperative patient-related/patient-reported outcomes and complications (e.g., re-operation, renal failure, sepsis, use of extracorporeal membrane oxygenation [ECMO], ventilation time, and length of stay [LOS]).

Where potential overlap between datasets was identified, we retained the study with the lower risk of bias or, if multiple studies with equivalent risk of bias were available, the one with the larger sample size. Overlap was considered when studies used the same registry and had overlapping data collection periods, and when the ratio of the smaller to the larger sample size suggested that approximately 20% or more of participants could potentially be included in both studies.

Furthermore, when multiple analyses from the same study examined the same outcome, only one analysis was included in the final synthesis. Priority was given to full-cohort analyses (rather than subgroup analyses), regardless of adjustment, and, where applicable, to analyses treating exposure as a continuous variable rather than categorical. When outcomes were reported using different summary measures or analytical models, we prioritized analyses using summary measures consistent with the majority of included studies and models with more extensive adjustment for confounders. This prioritization was applied consistently to avoid double counting and to improve comparability across studies.

Extracted data were presented in tables. When data were sufficiently homogeneous, we planned to conduct a random-effects meta-analysis using the metafor package [24] in R [25]. We intended to explore sources of heterogeneity through subgroup analyses (e.g., high vs. medium volume) or meta-regression. We also planned sensitivity analyses that excluded studies with a very high or high risk of bias.

### Reporting bias assessment

We planned to conduct reporting bias assessment using funnel plots for asymmetry and conducting Egger’s [26] and Begg’s [27] tests if more than ten studies per outcome were comparable in terms of exposure definition. Risk of bias in selection of the reported result was conducted using the ROBINS-E tool (domain number 7).

### Certainty assessment

The certainty of evidence was assessed using the Grading of Recommendations Assessment, Development, and Evaluation (GRADE) approach [28].

## Results

### Study selection

The previous version of the review included 10 studies reported in 11 publications. For the present update, electronic database searches retrieved 954 records from the initial search and 285 records from the updated search. After removal of 101 duplicate records, 1,138 records remained for screening.

Following title and abstract screening, 1,116 records were excluded. The remaining 22 full-text reports were retrieved and assessed for eligibility. Of these, 14 reports were excluded.

Additional 1785 records were identified through citation chasing, of which five reports were assessed for eligibility and all were included.

**Fig. 1:**
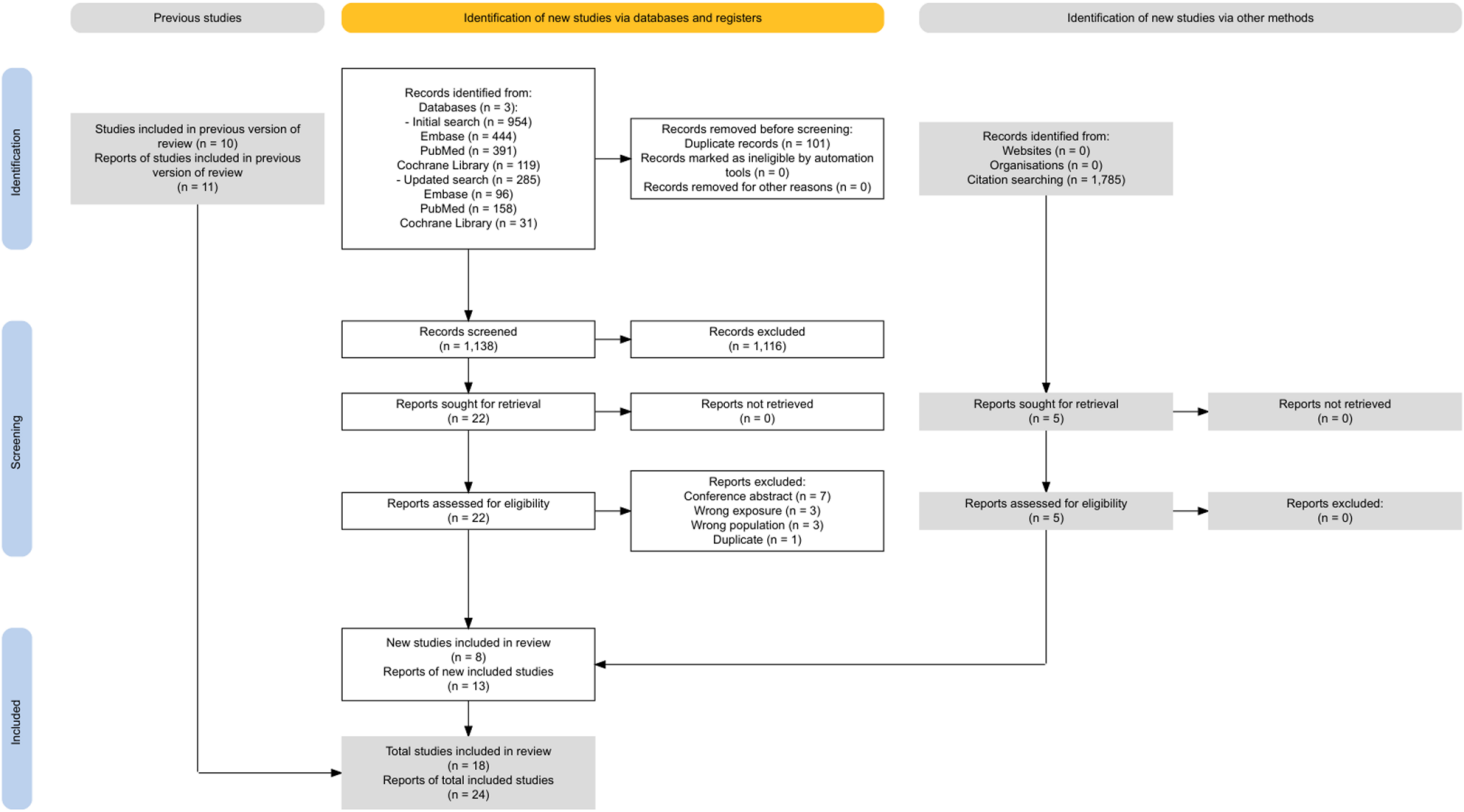
PRISMA flowchart

Overall, eight new studies (reported in 13 publications [29–41]) were included in the review. In total, the updated review comprises 18 studies, reported in 24 publications [18, 29–51]. The selection process is summarized in Fig. 1. An overview of excluded reports and the reasons for exclusion is provided in Supplementary File 2.

### Study characteristics

The 18 included studies were published between 2002 and 2025 and were based on data collected between 1982 and 2023 (Table 1). Most studies analyzed data from the United States (US) [29, 31–37, 41–46, 49, 50], with one study each analyzing data from the United Kingdom (UK) [30] and Japan [40], and five studies (six reports) using multinational data [18, 38, 39, 47, 48, 51]. Only two were prospective clinical studies, while the remaining 16 were retrospective analyses of data from administrative databases or clinical registries.

**Table 1:** Characteristics of included studies

Study characteristics								Patients’ characteristics					Exposure		
Study ID	Study type	Country	Data source	Data collection	No. of patients	No. of hospitals	No. of surgeons	Male (%)	Birth weight in kg	Low birth weight (%)	Age at Norwood (days)	Prematurity (%)	Hospital volume	Surgeon volume	Hospital type
Anderson 2016 [29]	Registry-based retrospective analysis	USA	Pediatric Health Information System (PHIS)	2004–2013	2880	35	NR	60.90%	3.10	NR	NR	NR	≤ 10/year 10-19 /year > 19/year as a continuous variable: with units of 10 operations/year	≤ 5/year 5-8.7/year > 8.7/year as a continuous variable: with units of 5 operations/ year	NI
Berry 2006 [42]	Registry-based retrospective analysis	USA	Kids’ inpatient database (KID)	1997 and 2000	1634	NR	NR	63%	NR	5%	NR	7%	Quartiles: Low Mid-low Mid-high High	NI	Non-teaching Teaching
Brown 2022 [30]	Registry-based retrospective analysis	UK	National Congenital Heart Diseases Audit (NCHDA)	April 1, 2000 - March 31, 2018	1399	10	NR	NR	NR	NR	NR	NR	as a continuous variable: scaled by 5 patients	NI	NI
SVR Trial 2012 [50], 2014 [36], 2018 [35, 37], 2022 [31]	Clinical prospective/ retrospective study	USA	Single Ventricle Reconstruction (SVR) Trial dataset	Enrollment between May 2005 and July 2008 with different follow-ups	544	NR	NR	62.50%	3.10	13.40%	6.8	11.20%	< 15/year > 15/year ≤ 15/year 16-20/year 21-30/year > 30/year	≤ 5/year > 5/year ≤ 5/year 6-10/year 11-15/year >15 as a continuous variable: per 1 unit increase/ year	NI
Chang 2002 [43]	Registry-based retrospective analysis	USA	Nationwide Inpatient Sample (NIS)	1988–1997	346	208	NR	NR	NR	NR	NR	NR	as a continuous variable	NI	NI
Chauhan 2024 [32]	Registry-based retrospective analysis	USA	Kids’ Inpatient Database (KID)	2016–2019	991	NR	NR	NR	NR	NR	NR	NR	Volume categories for pediatric cardiac surgery: ≤103/year 104–194/year ≥ 195/year	NI	NI
Checchia 2005 [44]	Registry-based retrospective analysis	USA	Pediatric Health Information System (PHIS)	1998–2001	801	29	87	NR	NR	NR	NR	NR	< 16 16-30 > 30 as a continuous variable	≤ 4 > 4 as a continuous variable	NI
Gong 2020 [34]	Registry-based retrospective analysis	USA	Kids’ Inpatient Database (KID)	2003, 2006, 2009, 2012, 2016	2872	NR	NR	62.10%	NR	NR	NR	NR	< 11/year 11-25/year > 25/year	NI	NI
Gutgesell 2002 [45]	Registry-based retrospective analysis	USA	University Health System Consortium (UHC)	1990–1999	1203	56	NR	NR	NR	NR	NR	NR	≤ 50/10 years > 50/10 years	NI	NI
Hirsch 2008 [46]	Registry-based retrospective analysis	USA	Kids’ inpatient database (KID)	2003	624	60	NR	63.10%	NR	NR	NR	NR	as a continuous variable	NI	Urban teaching Urban nonteaching Rural Unknown
Hornik 2012 [47]/ Pasquali 2012 [18]	Registry-based retrospective analysis	USA Canada	Society of Thoracic Surgeons Congenital Heart Surgery Database (STS-CHSD)	2000–2009	2557	53	111	58.20%	3.18	9.70%	NR	NR	≤ 10 11-20 ≥ 20 as a continuous variable: twofold decrease in hospital volume	≤ 5 6 − 10 > 10	NI
Karamlou 2010 [48]	Clinical prospective study	USA Canada	Congenital heart surgeons society (CHSS)	1994–2000	710	29	56	NR	NR	NR	NR	NR	Increased cases per year (per case)	Increased cases per year (per case)	NI
McHugh 2010 [49]/ Dean 2013 [33]	Registry-based retrospective analysis	USA	University HealthSystem Consortium (UHC)	1998–2007	1949	NR	NR	62%	NR	NR	NR	NR	McHugh: <20/10 years 20-64/10 years > 64/10 years Dean: >124/10 years ≤ 124/10 years	NI	NI
Schäfer 2025 [38]	Registry-based retrospective analysis	USA UK Canada	National Pediatric Cardiology–Quality Improvement Collaborative (NPC-QIC) registry	2016–2023	3397	69	NR	58.90%	3.10	3.30%	6	2.80%	≤ 5/year 6–10/year > 10/year	NI	NI
Welke 2023 [39]	Registry-based retrospective analysis	USA Canada	Society of Thoracic Surgeons Congenital Heart Surgery Database (STS-CHSD)	2017–2020	2453	90	NR	NR	NR	NR	NR	NR	as a continuous variable	NI	NI
Welke 2009 [51]	Registry-based retrospective analysis	USA Canada	Society of Thoracic Surgeons Congenital Heart Surgery Database (STS-CHSD)	2002–2006	1154	NR	NR	NR	NR	NR	NR	NR	Volume categories for pediatric cardiac surgery: <150/year 150–249/year 250–349/year ≥ 350/year as a continuous variable	NI	NI
Yoshimura 2023 [40]	Registry-based retrospective analysis	Japan	Japan Cardiovascular Surgery Database – Congenital (JCVSD-Congenital)	2013–2018	731	NR	NR	NR	NR	NR	NR	NR	Volume categories for pediatric cardiac surgery: 1–50/year 51–100/year 101–150/year ≥ 151/year	NI	NI
Zmora 2023 [41]	Registry-based retrospective analysis	USA	Pediatric Cardiac Care Consortium	1982–2011	1508	42	NR	62.90%	NR	NR	7	NR	as a continuous variable: 1-unit increase/per year	NI	NI

*NR* not reported, *NI* not investigated

Across 18 studies, the mean sample size was 1,542 patients. In nine studies reporting sex distribution, the median proportion of males was 62% (interquartile range [IQR]: 58–63). In three studies, age at the time of the Norwood procedure ranged from six to seven days. The proportion of patients with low birth weight (< 2.5 kg) ranged from 3% to 13% (data from four studies) with 3% to 11% being born prematurely (data from 3 studies). In the eleven studies reporting this information, the median number of hospitals was 53 (IQR: 15–179). The number of surgeons ranged from 56 to 111, with a median of 87 (data from three studies).

The relationship between patient outcomes and hospital volume was examined in all 18 studies. Additionally, five studies (reported in nine publications [18, 29, 35–37, 44, 47, 48, 50]) investigated surgeon volume, and two examined hospital teaching status [42, 46]. No studies on impact of centralization or regionalization were identified.

### Risk of bias assessments

Seventeen studies used observational data, while one study was based on data from the Pediatric Heart Network Single Ventricle Reconstruction (SVR) trial [31, 35–37, 50], a multicenter randomized controlled trial (RCT). However, participants in the SVR trial were not randomly assigned to hospitals or surgeons, meaning that the comparison is non-randomized. Therefore, all included studies were assessed with the ROBINS-E tool.

Across analyses of both primary and secondary outcomes (Supplementary file 3), ratings of some concerns were mainly driven by potential bias from post-exposure interventions, reflecting variability in postoperative care strategies and resource availability across hospitals. Ratings of high risk of bias were assigned when control for confounding was insufficient or when missing data or selection bias were present. Analyses judged to have a very high risk of bias made no attempt to control for confounding.

### Results of individual studies and narrative syntheses

The heterogeneity in how hospital and surgeon volumes were defined and handled in the analyses (e.g., categorization) was a key reason why a meta-analysis could not be performed for volume studies. Additionally, hospital types in the two included studies were defined differently, and while one study reported adjusted effect measures, the other presented unadjusted outcomes, limiting comparability. For these reasons, we summarized the findings narratively.

#### Synthesis of study results focusing on primary outcomes

##### Association between hospital volume and mortality-related outcomes

In the syntheses on hospital volume and mortality-related outcomes, of the 17 studies that met the eligibility criteria, three [32, 46, 51] were excluded due to substantial data overlap with other studies (an overview of the databases used across the studies with potential overlap, as well as the data collection periods, is provided in Supplementary File 4).

Across 14 studies included in the final synthesis [18, 29, 30, 33, 34, 38–45, 47–49] 20 of 26 analyses examining the association between hospital volume and mortality or survival were retained (Table 2). Of these, one analysis used overall CHS volume as the exposure of interest [40], with a higher number of Norwood procedures performed in higher CHS-volume categories during the study period. The remaining analyses focused on Norwood volume. Analyses excluded from the final synthesis can be found in Supplementary file 5.

**Table 2:** Results of studies examining association between hospital volume and mortality-related outcomes

Study ID	Outcome	Analysis/Model (adjustment)	Volume	Effect measure	Overall risk of bias
Anderson 2016 [29]	In-hospital mortality	Logistic regression (patient characteristics: sex, birth weight, prematurity, major co-morbid condition, dominant right ventricle, year of admission, long pre-operative time, insurance type and surgeon volume)	as a continuous variable: with units of 10 operations/year	OR 0.70 (CI: 0.56–0.89)	Some concerns
Berry 2006 [42]	In-hospital mortality	Logistic regression (teaching status, hospital volume, noncardiac structural anomaly, prematurity, low birth weight, aortic atresia, chromosomal anomaly	Quartiles: Low Mid-low Mid-high High	OR 3.1 (CI: 1.1–8.3) OR 2.0 (CI: 0.7–5.7) OR 1.0 (CI: 0.5–1.8) Ref.	Some concerns
Brown 2022 [30]	Long-term survival (median follow-up of 7.6 years)	Cox regression (Era, sex, additional cardiac risk factor, antenatal diagnosis, congenital noncardiac comorbidity, prematurity, low weight at the first cardiac procedure, acquired comorbidity at the first cardiac procedure, increased severity of illness at the first cardiac procedure)	as a continuous variable: scaled by 5 patients	HR 1.00 (CI: 0.97–1.03)	Some concerns
Chang 2002 [43]	In-hospital mortality	No model	as a continuous variable: 1988-1992 1993-1997	Correlation coefficients: *r* = -0.20 (*p* < 0.01) *r* = -0.31 (*p* < 0.01)	Very high risk
Checchia 2005 [44]	In-hospital mortality	Linear regression	as a continuous variable	Association of risk-unadjusted mortality r² = 0.18, *p* = 0.02	Very high risk
Checchia 2005 [44]	Time to death	No model	< 16/4 years 16-30/4 years > 30/4 years	Median (range) 19.2 (1–104) 5.4 (1–13) 7.8 (1–27) *p* > 0.05	Very high risk
Checchia 2005 [44]	28-day survival	No model	< 16/year 16-30/year > 30	48% 62% 71%	Very high risk
Gong 2020 [34]	In-hospital mortality	Logistic regression (gender; race/ethnicity; payer type; hospital region; income; No. of complex chronic conditions; comorbidities; LOS; ECMO)	< 11/year 11-25/year > 25/year	Ref. OR 0.73 (CI: 0.53-1.00, E = 1) OR 0.56 (CI: 0.39–0.80, E = 2.01)	High risk
Gutgesell 2002 [45]	In-hospital mortality	No model	≤ 50/10 years > 50/10 years	50% 40%	Very high risk
Hornik 2012 [47]/ Pasquali 2012 [18]	In-hospital mortality	Pasquali 2012/ Logistic regression (year of surgery, age, weight, sex, dominant ventricle, diagnosis of total anomalous pulmonary venous return, preoperative length of stay, the presence of any noncardiac/genetic abnormality, and preoperative shock, mechanical ventilatory or circulatory support, arrhythmia, complete atrioventricular block, neurologic deficit)	as a continuous variable: twofold decrease in hospital volume	OR 1.17 (CI: 1.01–1.35)	Some concerns
Karamlou 2010 [48]	5-year mortality	Hazard regression (Birth weight, age at operation, circulatory arrest time, ascending aortic dimension, reimplantation of the ascending aorta, shunt origin from the aorta)	Increased cases per year (per case)	Parameter estimate (SE) − 0.005 (± 0.01) *p* = 0.38	Some concerns
McHugh 2010 [49]/ Dean 2013 [33]	In-hospital mortality	McHugh 2010/ Logistic regression (female gender, prematurity, chromosomal anomality, endocardial cushion defect, double outlet right ventricle, era)	< 20/10 years 20-64/10 years > 64/10 years	OR 2.49 (CI: 1.51–4.07) OR 1.75 (CI: 1.23–2.49) Ref.	Some concerns
Schäfer 2025 [38]	30-day mortality	Logistic regression (prematurity, low birth weight (< 2.5 kg), ascending aorta size < 2 mm, intact or restrictive atrial septum, preoperative aortic valve regurgitation, moderate tricuspid valve regurgitation, and moderate ventricular dysfunction, surgical approach, age, weight, and sex)	≤ 5/year 6–10/year > 10/year	OR 1.15 (CI: 0.76–1.68) OR 1.13 (CI: 0.82–1.50) Ref.	High risk
Schäfer 2025 [38]	1-year mortality	Logistic regression (prematurity, low birth weight (< 2.5 kg), ascending aorta size < 2 mm, intact or restrictive atrial septum, preoperative aortic valve regurgitation, moderate tricuspid valve regurgitation, and moderate ventricular dysfunction, surgical approach, age, weight, and sex)	Low: ≤5/year Medium: 6–10/year High: >10/year	OR 1.41 (CI: 1.05–1.62) OR 1.30 (CI: 1.07–1.89) Ref.	High risk
Welke 2023 [39]	Operative mortality	Logistic regression (prematurity, prior cardiovascular operation, shock, renal failure, preoperative ventilator support, any other preoperative risk factor, any noncardiac congenital anatomic abnormality, chromosomal abnormality/syndrome categories with categories 3 to 5 collapsed together, age, and weight)	as a continuous variable (congenital heart surgery): 50/year 100/year 200/year 300/year 450/year	OR 3.46 (CrI: 1.98–5.74) OR 2.23 (CrI: 1.39–3.45) OR 1.19 (CrI: 0.69–1.88) OR 0.96 (CrI: 0.69–1.31) Ref.	Some concerns
Yoshimura 2023 [40]	Operative mortality	Logistic regression (J-STAT mortality category, age-weight category at time of surgery, urgency of surgery (elective, urgent, emergency, salvage), preoperative mechanical ventilation, preoperative inotropic agent use)	congenital heart surgery (Norwood cases during the study period): 1–50/year (15) 51–100/year (104) 101–150/year (168) ≥ 151/year (444)	Observed/Expected ratios: 2.75 (CI: 1.47–4.02) 1.86 (CI: 1.20–2.62) 1.72 (CI: 1.25–2.23) 0.88 (CI: 0.66–1.12)	Some concerns
Zmora 2023 [41]	In-hospital mortality	Logistic regression model (age (days) and weight z-score at the time of surgery, sex, chromosomal abnormality, and surgical era)	as a continuous variable: 1-unit increase/year	OR 0.955 (CI: 0.935–0.976)	Some concerns
Zmora 2023 [41]	90-day mortality	Logistic regression (age (days) and weight z-score at the time of surgery, sex, chromosomal abnormality, and surgical era)	as a continuous variable: 1-unit increase/year	OR 0.96 (CI: 0.93–0.99)	Some concerns
Zmora 2023 [41]	1-year mortality	Logistic regression model (age (days) and weight z-score at the time of surgery, sex, chromosomal abnormality, and surgical era)	as a continuous variable: 1-unit increase/year	OR 0.97 (CI: 0.95–0.99)	Some concerns
Zmora 2023 [41]	3-year mortality	Logistic regression model (age (days) and weight z-score at the time of surgery, sex, chromosomal abnormality, and surgical era)	as a continuous variable: 1-unit increase/year	OR 0.97 (CI: 0.96–0.99)	Some concerns

*OR *odds ratio, *CI* 95% confidence interval, *HR *hazard ratio, *SD *standard deviation, *r *correlation coefficient, *E* E-value for unmeasured confounding, *LOS *length of stay, *ECMO* extracorporeal membrane oxygenation, *SE *standard error, *CrI* 95% credible interval, *J-STAT* Japan Society of Thoracic Surgeons-European Association for Cardiothoracic Surgery Congenital Heart Surgery, *Ref*. reference

Short-term mortality outcomes were examined across 12 studies [18, 29, 34, 38–40, 42–45, 49], with some studies analyzing more than one outcome (e.g., in-hospital and 90-day mortality [41]), resulting in a total of 15 analyses. Of these, 14 analyses reported results in favor of higher hospital volume, including ten with statistically significant findings, while an analysis by Checchia et al. [25] on median time to death reported non-statistically significant results favoring lower volume.

Long-term mortality outcomes were examined in five analyses of four studies [30, 38, 41, 48]. Four analyses reported results supporting a hospital volume-outcome relationship, three of which were statistically significant, while one analysis [30] reported no association.

##### Association between surgeon volume and mortality-related outcomes

Eight of the nine analyses examining surgeon volume and mortality or survival were included in the final synthesis. Details of the excluded analysis are provided in Supplementary file 5.

Three short-term outcomes (in-hospital mortality, 28-day survival and death or transplantation before stage II) were examined in five analyses across four studies [29, 35, 44, 47] (Table 3). Three analyses favored higher surgeon volume [35, 44, 47], one of which reached statistical significance, while one analysis reported non-statistically significant findings in the opposite direction [29]. One analysis of Checchia and colleagues [44] reported non-significant p-values without providing correlation coefficients. For long-term outcomes, all three analyses (death or cardiac transplant at 3 and 6 years and 5-year mortality) conducted in two studies favored higher surgeon volume [36, 37, 48], with two analyses reaching statistical significance.

**Table 3:** Results of studies examining association between surgeon volume and mortality-related outcomes

Study ID	Outcome	Analysis/Model (adjustment)	Volume	Effect measure	Overall risk of bias
Anderson 2016 [29]	In-hospital mortality	Logistic regress ion (patient characteristics and hospital volume)	as a continuous variable: with units of 5 operations/year	OR 1.02 (CI: 0.87–1.20)	Some concerns
Chechia 2005 [44]	In-hospital mortality	Linear regression	as a continuous variable	r² = NR, *p* = 0.312	Very high risk
Chechia 2005 [44]	28-day survival	No model	≤ 4/4 years > 4/4 years	49% 69% *p* = 0.13	Very high risk
Hornik 2012 [47]/ Pasquali 2012 [18]	In-hospital mortality	Hornik 2012/ Logistic regression (patient characteristics, hospital volume)	≤ 5 6 − 10 >10	OR 1.47 (CI: 1.01–2.15) OR 1.26 (CI: 0.88–1.78) Ref.	Some concerns
Karamlou 2010 [48]	5-year mortality	Hazard regression (Birth weight, age at operation, circulatory arrest time, ascending aortic dimension, reimplantation of the ascending aorta, shunt origin from the aorta)	Increased cases per year (per case)	−0.004 ± 0.007 (*p* = 0.49) [parameter estimate ± SE]	Some concerns
SVR Trial/ Jean-St-Michel 2018 [35]	Death or cardiac transplant before stage II	Multivariable parametric hazard model (Shunt type (MBTS vs. RVPA conduit), post-Norwood infectious disease complication, right ventricular end-diastolic long-axis dimension (per cm))	≤ 5/year > 5/year	PE 1.10 (CI: 0.24–1.97; *p* = 0.01) Ref.	High risk
SVR Trial/ Newburger 2014 [36]	Death or cardiac transplant at 3 years	Logistic regression (aortic stenosis/mitral stenosis, obstructed pulmonary venous drainage, genetic syndrome, moderate/severe, open sternum on day of Norwood procedure, ECMO used in operating room, when birth weight < 2.5 kg, every 0.5 kg increase, When birth weight ≥ 2.5 kg, every 1 kg increase)	≤ 5/year > 5/year	OR 1.73 (CI: 1.05–2.85) Ref.	Some concerns
SVR Trial/ Newburger 2018 [37]	Death or cardiac transplant at 6 years	Multivariable Cox Regression (Shunt × Time interaction; RVPAS vs. MBTS before stage II procedure; RVPAS vs. MBTS, stage II to Fontan procedure; RVPAS vs. MBTS, after Fontan procedure; Total anomalous pulmonary venous return; Genetic syndrome; Pre-Norwood moderate/severe tricuspid regurgitation; ECMO used in operating room)	as a continuous variable: 1 unit increase/year	HR 0.95 (95% CI: 0.93–0.98)	Some concerns

*OR *odds ratio, *PE *parameter estimate, *Ref*. reference, *SVR *Single Ventricle Reconstruction, *MBTS *modified Blalock-Taussig shunt, *RVPA(S) *right ventricle to pulmonary artery (shunt), *ECMO *extracorporeal membrane oxygenation, *SE *standard error, *HR *hazard ratio, *NR* not reported

##### Association between hospital teaching status and mortality-related outcomes

Two studies evaluated the association between hospital teaching status and in-hospital mortality [42, 46] (Table 4). One study [42] reported a statistically significant association, with non-teaching hospitals showing higher mortality compared to teaching hospitals. The other study [46] presented unadjusted mortality rates across hospital types, reporting higher mortality in urban non-teaching and rural hospitals compared to urban teaching hospitals.

**Table 4:** Results of studies examining association between hospital type and mortality-related outcomes

Study ID	Outcome	Model (adjustment)	Hospital type	Effect measure	Overall risk of bias
Berry 2006 [42]	In-hospital mortality	Logistic regression (teaching status, hospital volume, noncardiac structural anomaly, prematurity, low birth weight, aortic atresia, chromosomal anomaly)	Non-teaching Teaching	OR 2.6 (CI: 1.3–5.3) Ref.	Some concerns
Hirsch 2008 [46]	In-hospital mortality	No model	Urban teaching Urban nonteaching Rural Unknown	24.4% 32.2% 34.0% 26.6%	Very high risk

*OR *odds ratio, *Ref*. reference

#### Synthesis of study results focusing on secondary outcomes

A total of 17 analyses across six studies examining the association between hospital or surgeon volume and secondary outcomes were included in the final synthesis (analyses excluded from the final synthesis can be found in Supplementary file 5). No study examined the association between hospital teaching status and secondary outcomes.

##### Association between hospital volume and secondary outcomes

After exclusion of the renal failure analysis by Chamberlain et al. [31] due to complete data overlap with an earlier analysis by Tabbutt et al. [50] using SVR trial data, 13 analyses of hospital volume and secondary outcomes from six studies [29, 34, 38, 39, 44, 50] were included in the final synthesis (Supplementary file 6, Table 1).

LOS was the most frequently assessed secondary outcome in relation to hospital volume, with five analyses across five studies [29, 34, 39, 44, 50]. Results of four analyses across four studies [29, 39, 44, 50] favored higher hospital volume, with two analyses reporting statistically significant results. One analysis conducted by Gong et al. [34] reported non-statistically significant results in the opposite direction.

Other secondary outcomes examined in eight analyses across four studies [34, 38, 39, 50] included: ECMO use [34], failure to rescue [39], length of ventilation [50], time to first extubation [50], major complications [39], need for unplanned catheterization [38], renal failure [50], and sepsis [50]. Seven of the eight analyses favored higher volume, with five reporting statistically significant results. In contrast, the statistically significant results of the analysis on time to first extubation favored the lowest hospital volume category [50].

##### Association between surgeon volume and secondary outcomes

Four analyses across two studies [29, 50] examined the association between surgeon volume and secondary outcomes (Supplementary file 6, Table 2). An analysis by Anderson et al. [29] favored the highest surgeon volume category, but did not reach statistical significance. Two analyses by Tabbutt et al. [50] favored the highest surgeon volume category for time to first extubation and ventilation and were statistically significant. In contrast, another analysis by Tabbutt et al. [50] on renal failure reported non-statistically significant results favoring the lowest surgeon category.

### Reporting bias assessment

A funnel plot or other tests for publication bias could not be conducted due to lack of sufficient number of studies/analyses with comparable definitions of exposure. Given these limitations, publication bias was not formally assessed and therefore remains undetected. Furthermore, we did not identified substantial concerns regarding risk of bias in selection of the reported result (Supplementary file 3, Tables 1 and 2, domain 7) while using ROBINS-E.

### Certainty of evidence

According to GRADE, the certainty of the evidence for all analyzed critical primary outcomes was very low (Table 5). The certainty was downgraded mainly due to risk of bias and indirectness.

**Table 5:** Summary of findings and certainty of evidence (GRADE)

Exposure	Outcome	No. of studies (analyses)	No. of participants	Impact	Certainty (reasons for downgrading)	Importance
Hospital volume	Mortality** (≤ 90 days)** [18, 29, 33, 34, 38–45, 47, 49]	12 (15)	22,331	All but one analysis reported results in favor of higher-volume hospitals	Very low ⊕◯◯◯ (-2 for RoB^a^ -1 for inconsistency^b^ -1 for indirectness^c^)	Critical
	Long-term mortality [30, 38, 41, 48]	4 (5)	7,014	4 analyses support volume-outcome relationship; 1 analysis reported no relationship	Very low ⊕◯◯◯ (-2 for RoB^d^ -1 for inconsistency^b^ -1 for indirectness^c^ -1 for imprecision^e^)	Critical
Surgeon volume	Mortality** (< Stage II)** [29, 35, 44, 47]	4 (5)	6,361	Inconsistent results: 3 analyses support volume-outcome relationship; 1 reported non-statistically significant results in favor of lower-volume surgeons; 1 analysis reported non-significant p-values without providing correlation coefficients	Very low ⊕◯◯◯ (-2 for RoB^f^ -1 for inconsistency^b^ -1 for indirectness^c^ -1 for imprecision^e^)	Critical
	Long-term mortality [36, 48]	2 (3)	1,237	All analyses support volume-outcome relationship	Very low ⊕◯◯◯ (-1 for RoB^g^ -1 for indirectness^c^ -1 for imprecision^h^)	Critical
Hospital teaching status	In-hospital mortality [42, 46]	2 (2)	2,258	Both analyses support the relationship between hospital teaching status and lower mortality	Very low ⊕◯◯◯ (-2 for RoB^i^ -1 for indirectness^j^)	Critical

*RoB *risk of bias

^a^ ROBINS-E: 7 analyses with high or very high risk of bias

^b^ Results show different directions of effect/no effect

^c^ Studies used different volume definitions; all studies were conducted using data from high-income countries

^d^ Robins-E: 1 analysis with high risk of bias

^e^ 95% confidence interval in some analyses includes null effect

^f^ ROBINS-E: 3 analyses with high or very high risk of bias

^g^ Robins-E: All analyses were assessed to have some concerns regarding risk of bias

^h^ Non statistically significant p value of the parameter estimate in one analysis

^i^ ROBINS-E: 1 analysis with very high risk of bias

^j^ Studies used different hospital status definitions

## Discussion

This updated systematic review synthesized evidence from 18 studies (24 publications) examining the relationship between hospital volume, surgeon volume, hospital teaching status, and patient outcomes following the Norwood procedure for HLHS. Compared with the 2014 review, eight new studies and 13 additional publications were identified, with the geographic scope expanding beyond the US and Canada to include data Japan, and the UK.

### General interpretation of the results in the context of other evidence

When interpreting these findings, it is important to consider the potential role of institutional factors that were not accounted for across the included studies. These may include the availability of dedicated pediatric intensive care units, the specific perioperative and interstage management protocols followed, staffing, and other institutional resources. If such factors are associated with both the exposures of interest and outcomes of Norwood procedure, their omission may have contributed to residual confounding. For example, evidence from pancreatic surgery suggests that unmeasured confounding may lead to overestimation of volume-outcome associations [52]. Thus, the observed associations discussed below may partly reflect unmeasured differences in institutional organization and resources rather than the exposures of interest alone.

#### Hospital volume

Overall, the findings of this update indicate a hospital volume-outcome relationship for most mortality outcomes following the Norwood procedure. In the original review, most analyses of early mortality reported lower mortality in higher-volume hospitals. Evidence on longer-term mortality in the original review was limited to a single analysis, which also favored higher-volume centers. In this update, all new analyses of early mortality showed effect estimates in favor of higher-volume hospitals. However, findings for long-term mortality were less consistent, with one analysis reporting no association [30]. These findings suggest that the association between hospital volume and mortality is most pronounced in the early postoperative period. This is in line with the findings of the subgroup analyses in the study by Zmora et al. [41], where, after exclusion of early deaths (< 90 days), the previously observed effect estimates favoring higher-volume hospitals were no longer evident for either 1- or 3-year mortality.

Our findings on early mortality are in line with the results reported for pediatric cardiac surgery in an earlier SR [53]. It is important to note, however, that some studies were also included in the current SR. Taken together with results from other primary studies from the US and Europe [54, 55], these findings suggest that hospital volume effects may also be present in other CHS. However, evidence for other types of pediatric surgery is inconsistent, suggesting the association between hospital volume and early mortality may be indication-specific or less evident beyond CHS. For example, results of an SR on gastroschisis repair suggest that higher hospital volume may reduce in-hospital mortality [56], whereas results of an SR on congenital diaphragmatic hernia repair are inconclusive [57]. Furthermore, two primary studies on esophageal atresia with or without tracheoesophageal fistula reported no statistically significant relationship [58, 59].

Consistent with the observed findings for mortality outcomes, most analyses of secondary outcomes also tended to favor higher-volume hospitals. For LOS, four of five analyses favored hospitals with higher procedural volumes, whereas one analysis favored lower-volume hospitals [34]. These two opposing trends can be explained by competing mechanisms influencing LOS. On the one hand, higher-volume hospitals may have shorter LOS due to greater perioperative care efficiency [39], leading to improved surgery outcomes and a lower occurrence of complications [60]. On the other hand, longer LOS may reflect the treatment of higher-complexity cases in high-volume hospitals [61] that require prolonged postoperative inpatient care [62]. Evidence for other secondary outcomes was limited to one analysis per outcome but the results generally pointed in a direction consistent with improved postoperative outcomes in higher-volume hospitals.

#### Surgeon volume

In contrast to hospital volume, results for surgeon volume were mixed for early mortality-related outcomes [29, 35, 44, 47], but more consistently favored higher surgeon volume for long-term outcomes, including 3- and 6-year transplantation-free survival and 5-year mortality [36, 37, 48]. This may reflect the role of surgical technical skills, which may have a more pronounced long-term influence on survival than hospital volume. For example, suboptimal surgical technique resulting in intraprocedural cardiac arrest might not lead to immediate death if the overall team experience and institutional resources are sufficient to rescue the patient [63]. However, as the patient progresses through the interstage period and beyond, the anatomical and physiological consequences of the initial surgery may become more apparent.

Evidence addressing surgeon volume and mortality beyond the Norwood procedure seems limited. The previously mentioned SR [53] included only a single study in pediatric cardiac surgery, which reported a statistically significant association between higher surgeon volume and lower in-hospital mortality [64]. A more recent primary study of outcomes after the arterial switch operation suggests a similar impact [65]. Outside the context of CHS, evidence examining the relationship between surgeon volume and mortality is likewise limited, with two SRs noting that they did not identify any evidence addressing surgeon volume [56, 57].

With respect to secondary outcomes, we identified only one new study that examined the impact of surgeon volume, which reported shorter LOS among patients treated by higher-volume surgeons [29]. One possible contributing factor described in the literature is greater operative efficiency associated with increased surgical experience. For instance, in arterial switch operations, higher surgeon volume has been shown to predict shorter cardiopulmonary bypass and cross-clamp times, both major determinants of faster recovery and shorter LOS [65]. Beyond this newly identified study, findings from the original review suggest that higher surgeon volume may be associated with shorter duration of mechanical ventilation and earlier extubation, whereas results for renal failure favored lower surgeon volume [50].

#### Hospital type

In contrast to the evidence on hospital and surgeon volume, no new studies on hospital teaching status and mortality were identified beyond those in the original review. As a result, the evidence remains limited to two studies using data from 1997 to 2003, both of which showed lower mortality in teaching hospitals. We are not aware of other evidence syntheses or primary studies with a comparative design examining the association between hospital type and mortality in pediatric patients. Beyond mortality, no studies examining secondary outcomes in relation to hospital teaching status were included in this updated review.

### Limitations of the evidence

Several characteristics of the identified evidence may limit the generalizability of the findings of this SR update. First, 16 of 18 studies used data from the US, either exclusively or in combination with data from Canada or the UK, whereas only two were conducted entirely outside North America. As a result, caution is warranted when extrapolating these findings to other settings, as differences in socioeconomic context and organization of care may limit comparability across healthcare systems.

Second, in all but two studies, the analyzed data originated from administrative databases or clinical registries. Specifically, administrative databases are prone to coding inaccuracies and often lack detailed clinical information, which can lead to misclassification of procedures and outcomes [66]. Reliance on such data also increases the risk of overlapping patient populations across studies [67]. Although we excluded five analyses using identical data sources, potential overlap across different databases could not be fully assessed because information on participating hospitals was often unavailable. Greater transparency regarding data sources and coverage would strengthen future SRs using routinely collected data.

Third, the lack of standardized volume definitions across studies precluded meta-analysis and limited the ability to identify clinically meaningful cut-offs. Future research should establish and validate standardized definitions of surgeon and hospital volume.

Fourth, the included studies generally did not use causal inference methods. Approaches such as target trial emulation [68] could strengthen causal interpretation and reduce confounding. The study by Madenci et al. [69] illustrates how this framework can be applied to volume-outcome research.

Fifth, the data analyzed in the included studies span more than four decades (1982–2023), during which surgical techniques, perioperative management, and the organization of care for HLHS underwent substantial changes [70–73]. These temporal developments may have influenced Norwood outcomes independently of hospital or surgeon volume and hospital teaching status.

Finally, no studies focused on patient-reported outcomes such as health-related quality of life, despite the availability of a congenital heart disease core outcome set that recommends assessing such outcomes [74]. Future research should incorporate patient-reported outcomes to determine whether hospital or surgeon volume, or other exposures of interest, affect outcomes beyond routinely examined clinical metrics.

### Limitations of review processes

Despite comprehensive database searches and citation chasing, exclusion of conference abstracts and grey literature may have resulted in missed eligible studies. In addition, due to resource constraints, data extraction was performed by one reviewer and checked by a second, an approach that may not have detected all inaccuracies compared with full independent duplicate extraction. Finally, although we attempted to assess potential overlap between study populations, incomplete information on participating centers and cross-registry coverage limited this assessment, and duplication across data sources cannot be ruled out.

### Implications for clinical practice and policy

Overall, despite the very low quality of evidence our findings support consideration of service organization strategies that concentrate Norwood care in hospitals with sufficient experience and resources. However, the available evidence is insufficient to define a generalizable minimum-volume threshold. Furthermore, our findings do not allow conclusions about whether volume or hospital teaching status themselves influence clinical outcomes, or whether the results reflect other aspects of care. Any actions, whether in clinical practice or policy, should therefore be planned and implemented cautiously, informed by local healthcare contexts and by evidence from studies using designs and analytical approaches that support causal interpretation, and accompanied by ongoing evaluation of outcomes, access, and equity.

## Conclusions

This updated SR synthesized evidence from 18 studies reported in 24 publications examining the relationship between hospital volume, surgeon volume, hospital teaching status, and patient-related outcomes after the Norwood procedure. Overall, analyses of hospital volume more consistently favored higher-volume hospitals for early mortality than for long-term mortality. In contrast, analyses of surgeon volume showed less consistent findings for early mortality-related outcomes, whereas all analyses of long-term mortality and transplant-free survival favored higher surgeon volume. Findings for secondary outcomes were mostly based on single analyses and were mixed, although the majority favored higher hospital or surgeon volume. Evidence on hospital teaching status is limited to two older studies which report improved in-hospital mortality in teaching hospitals.

In conclusion, although the available evidence points toward both a volume-outcome relationship and a relationship with hospital teaching status for the Norwood procedure, the certainty of this evidence is very low, which limits the extent to which these findings can inform policy and practice. Future efforts should focus on strengthening methodological approaches in volume-outcome research that support estimation of the causal effect and contribute to the identification of clinically meaningful volume thresholds, thereby strengthening the evidence base for guiding clinical practice and policy.

## Supplementary Information

Supplementary Material 1.

Supplementary Material 2.

Supplementary Material 3.

Supplementary Material 4.

Supplementary Material 5.

Supplementary Material 6.

## Data Availability

All data generated or analyzed during this study are included in this published article and its supplementary information files. Additional details regarding methodology and data are available upon reasonable request from the corresponding author.

## Abbreviations

CHS: Congenital heart surgery
COS: Core outcome set
ECMO: Extracorporeal membrane oxygenation
GRADE: Grading of Recommendations Assessment, Development, and Evaluation
HLHS: Hypoplastic left heart syndrome
HR: Hazard ratio
IQR: Interquartile range
LOS: Length of stay
OR: Odds ratio
PRISMA: 2020–Preferred Reporting Items for Systematic Reviews and Meta–Analyses 2020
RCT: Randomized controlled trial
RoB2: Cochrane Risk of Bias 2 tool
ROBINS: E–Risk Of Bias In Non–randomized Studies of Exposures
SR: Systematic review
SVR trial: The Pediatric Heart Network Single Ventricle Reconstruction trial
UK: United Kingdom
US: United States

## References

[1] Mohanty SR, Patel A, Kundan S, Radhakrishnan HB, Rao SG. Hypoplastic left heart syndrome: current modalities of treatment and outcomes. Indian J Thorac Cardiovasc Surg. 2021;37(Suppl 1):26–35. 33584025 10.1007/s12055-019-00919-7 PMC7859121

[2] Ohye RG, Sleeper LA, Mahony L, Newburger JW, Pearson GD, Lu M, et al. Comparison of shunt types in the Norwood procedure for single-ventricle lesions. N Engl J Med. 2010;362(21):1980–92. 20505177 10.1056/NEJMoa0912461 PMC2891109

[3] Roeleveld PP, Axelrod DM, Klugman D, Jones MB, Chanani NK, Rossano JW, et al. Hypoplastic left heart syndrome: from fetus to fontan. Cardiol Young. 2018;28(11):1275–88. 30223915 10.1017/S104795111800135X

[4] Ramachandran P, King E, Nebbia A, Beekman RH, Anderson JB. Variability of antithrombotics use in patients with hypoplastic left heart syndrome and its variants following first- and second-stage palliation surgery: a national report using the National Pediatric Cardiology Quality Improvement Collaborative registry. Cardiol Young. 2017;27(4):731–8. 27981915 10.1017/S1047951116001189

[5] Meza JM, Blackstone EH, Argo MB, Thuita L, Lowry A, Rajeswaran J, et al. A dynamic Norwood mortality estimation: Characterizing individual, updated, predicted mortality trajectories after the Norwood operation. JTCVS Open. 2023;14:426–40. 37425467 10.1016/j.xjon.2023.04.010 PMC10329031

[6] John MM, McKenzie ED. Norwood procedure: How I do it. JTCVS Tech. 2020;4:205–7. 34318015 10.1016/j.xjtc.2020.08.026 PMC8305239

[7] Hornik CP, He X, Jacobs JP, Li JS, Jaquiss RD, Jacobs ML, et al. Complications after the Norwood operation: an analysis of The Society of Thoracic Surgeons Congenital Heart Surgery Database. Ann Thorac Surg. 2011;92(5):1734–40. 21937021 10.1016/j.athoracsur.2011.05.100 PMC3246682

[8] Bezerra RF, Pacheco JT, Volpatto VH, Franchi SM, Fitaroni R, da Cruz DV, et al. Extracorporeal Membrane Oxygenation After Norwood Surgery in Patients With Hypoplastic Left Heart Syndrome: A Retrospective Single-Center Cohort Study From Brazil. Front Pediatr. 2022;10:813528. 35311057 10.3389/fped.2022.813528 PMC8926323

[9] McHugh KE, Pasquali SK, Hall MA, Scheurer MA. Impact of postoperative complications on hospital costs following the Norwood operation. Cardiol Young. 2016;26(7):1303–9. 26714435 10.1017/S1047951115002498 PMC6383204

[10] Birkmeyer JD, Siewers AE, Finlayson EV, Stukel TA, Lucas FL, Batista I, et al. Hospital volume and surgical mortality in the United States. N Engl J Med. 2002;346(15):1128–37. 11948273 10.1056/NEJMsa012337

[11] Vemulapalli S, Carroll JD, Mack MJ, Li Z, Dai D, Kosinski AS, et al. Procedural Volume and Outcomes for Transcatheter Aortic-Valve Replacement. N Engl J Med. 2019;380(26):2541–50. 30946551 10.1056/NEJMsa1901109

[12] Bekelis K, Connolly ID, Do HM, Choudhri O. Operative volume and outcomes of cerebrovascular neurosurgery in children. J Neurosurg Pediatr. 2016;18(5):623–8. 27494548 10.3171/2016.5.PEDS16137

[13] Luft HS, Hunt SS, Maerki SC. The volume-outcome relationship: practice-makes-perfect or selective-referral patterns? Health Serv Res. 1987;22(2):157–82. 3112042 PMC1065430

[14] Luft HS, Bunker JP, Enthoven AC. Should operations be regionalized? The empirical relation between surgical volume and mortality. N Engl J Med. 1979;301(25):1364–9. 503167 10.1056/NEJM197912203012503

[15] Ghandour HZ, Vervoort D, Welke KF, Karamlou T. Regionalization of congenital cardiac surgical care: what it will take. Curr Opin Cardiol. 2022;37(1):137–43. 34654032 10.1097/HCO.0000000000000940

[16] Pieper D, Mathes T, Asfour B. A systematic review of the impact of volume of surgery and specialization in Norwood procedure. BMC Pediatr. 2014;14:198. 25096305 10.1186/1471-2431-14-198 PMC4127072

[17] Page MJ, McKenzie JE, Bossuyt PM, Boutron I, Hoffmann TC, Mulrow CD, et al. The PRISMA 2020 statement: an updated guideline for reporting systematic reviews. BMJ. 2021;372:n71. 33782057 10.1136/bmj.n71 PMC8005924

[18] Pasquali SK, Jacobs JP, He X, Hornik CP, Jaquiss RD, Jacobs ML, et al. The complex relationship between center volume and outcome in patients undergoing the Norwood operation. Ann Thorac Surg. 2012;93(5):1556–62. 22014746 10.1016/j.athoracsur.2011.07.081 PMC3334400

[19] Haddaway NR, Grainger MJ, Gray CT, Citationchaser. A tool for transparent and efficient forward and backward citation chasing in systematic searching. Res Synth Methods. 2022;13(4):533–45. 35472127 10.1002/jrsm.1563

[20] Ouzzani M, Hammady H, Fedorowicz Z, Elmagarmid A. Rayyan-a web and mobile app for systematic reviews. Syst Rev. 2016;5(1):210. 27919275 10.1186/s13643-016-0384-4 PMC5139140

[21] Higgins JPT, Morgan RL, Rooney AA, Taylor KW, Thayer KA, Silva RA, et al. A tool to assess risk of bias in non-randomized follow-up studies of exposure effects (ROBINS-E). Environ Int. 2024;186:108602. 38555664 10.1016/j.envint.2024.108602 PMC11098530

[22] Gutzeit M, Rauh J, Kähler M, Cederbaum J. Modelling Volume-Outcome Relationships in Health Care. Stat Med. 2025;44(6):e10339. 40042399 10.1002/sim.10339

[23] Sterne JA, Savović J, Page MJ, Elbers RG, Blencowe NS, Boutron I et al. RoB 2: a revised tool for assessing risk of bias in randomised trials. BMJ. 2019;366:l4898. 10.1136/bmj.l4898 31462531

[24] Viechtbauer W. Conducting Meta-Analyses in R with the metafor Package. J Stat Softw. 2010;36(3):1–48.

[25] R Core Team. R: A language and environment for statistical computing. R Foundation for Statistical Computing, Vienna, Austria. https://www.R-project.org/.

[26] Egger M, Davey Smith G, Schneider M, Minder C. Bias in meta-analysis detected by a simple, graphical test. BMJ. 1997;315(7109):629–34. 9310563 10.1136/bmj.315.7109.629 PMC2127453

[27] Begg CB, Mazumdar M. Operating characteristics of a rank correlation test for publication bias. Biometrics. 1994;50(4):1088–101. 7786990

[28] Schünemann H, Brożek J, Guyatt G, Oxman A. The GRADE handbook. Cochrane Collaboration London, UK; 2013. https://gdt.gradepro.org/app/handbook/handbook.html.

[29] Anderson BR, Ciarleglio AJ, Cohen DJ, Lai WW, Neidell M, Hall M, et al. The Norwood operation: Relative effects of surgeon and institutional volumes on outcomes and resource utilization. Cardiol Young. 2016;26(4):683–92. 26169083 10.1017/S1047951115001031 PMC4713384

[30] Brown KL, Huang Q, Hadjicosta E, Seale AN, Tsang V, Anderson D et al. Long-term survival and center volume for functionally single-ventricle congenital heart disease in England and Wales. J Thorac Cardiovasc Surg. 2023;166(2):306–16.e3. 10.1016/j.jtcvs.2022.11.018 36535820

[31] Chamberlain RC, Andersen ND, McCrary AW, Hornik CP, Hill KD. Postoperative Renal Failure, Shunt Type, and Mortality After Norwood Palliation. Ann Thorac Surg. 2022;113(6):2046–53. 34534529 10.1016/j.athoracsur.2021.08.019 PMC8920938

[32] Chauhan D, Mehaffey JH, Hayanga JWA, Udassi JP, Badhwar V, Mascio CE. Volume Alone Does Not Predict Quality Outcomes in Hospitals Performing Pediatric Cardiac Surgery. Ann Thorac Surg. 2024;117(6):1187–93. 38290594 10.1016/j.athoracsur.2024.01.006

[33] Dean PN, McHugh K, Hillman DG, Conaway MR, Gutgesell H. Effects of race, ethnicity and gender on surgical mortality for hypoplastic left heart syndrome. J Am Coll Cardiol. 2013;61(10):E431. 10.1007/s00246-013-0723-3 PMC4023351 23722968

[34] Gong CL, Song AY, Horak R, Friedlich PS, Lakshmanan A, Pruetz JD, et al. Impact of Confounding on Cost, Survival, and Length-of-Stay Outcomes for Neonates with Hypoplastic Left Heart Syndrome Undergoing Stage 1 Palliation Surgery. Pediatr Cardiol. 2020;41(5):996–1011. 32337623 10.1007/s00246-020-02348-5

[35] Jean-St-Michel E, Meza JM, Maguire J, Coles J, McCrindle BW. Survival to Stage II with Ventricular Dysfunction: Secondary Analysis of the Single Ventricle Reconstruction Trial. Pediatr Cardiol. 2018;39(5):955–66. 29520465 10.1007/s00246-018-1845-4

[36] Newburger JW, Sleeper LA, Frommelt PC, Pearson GD, Mahle WT, Chen S, et al. Transplantation-free survival and interventions at 3 years in the single ventricle reconstruction trial. Circulation. 2014;129(20):2013–20. 24705119 10.1161/CIRCULATIONAHA.113.006191 PMC4029928

[37] Newburger JW, Sleeper LA, Gaynor JW, Hollenbeck-Pringle D, Frommelt PC, Li JS, et al. Transplant-Free Survival and Interventions at 6 Years in the SVR Trial. Circulation. 2018;137(21):2246–53. 29437119 10.1161/CIRCULATIONAHA.117.029375 PMC5963989

[38] Schäfer M, McFarland C, Amula V, Truong D, Lambert LM, Griffiths ER, et al. Volume-Outcome Relationship of Norwood Procedures: Insights from the National Pediatric Cardiology-Quality Improvement Collaborative Database. Ann Thorac Surg. 2025;119(5):1045–52. 39864776 10.1016/j.athoracsur.2025.01.007

[39] Welke KF, Karamlou T, O’Brien SM, Dearani JA, Tweddell JS, Kumar SR, et al. Contemporary Relationship Between Hospital Volume and Outcomes in Congenital Heart Surgery. Ann Thorac Surg. 2023;116(6):1233–9. 37652353 10.1016/j.athoracsur.2023.08.006

[40] Yoshimura N, Hirata Y, Inuzuka R, Tachimori H, Hirano A, Sakurai T, et al. Effect of procedural volume on the outcomes of congenital heart surgery in Japan. J Thorac Cardiovasc Surg. 2023;165(4):1541–e503. 35963799 10.1016/j.jtcvs.2022.06.009

[41] Zmora R, Spector L, Bass J, Thomas A, Knight J, Lakshminarayan K, et al. Procedure-Specific Center Volume and Mortality After Infantile Congenital Heart Surgery. Ann Thorac Surg. 2023;116(3):525–31. 37100164 10.1016/j.athoracsur.2023.04.020 PMC10524585

[42] Berry JG, Cowley CG, Hoff CJ, Srivastava R. In-hospital mortality for children with hypoplastic left heart syndrome after stage I surgical palliation: teaching versus nonteaching hospitals. Pediatrics. 2006;117(4):1307–13. 16585328 10.1542/peds.2005-1544

[43] Chang RK, Chen AY, Klitzner TS. Clinical management of infants with hypoplastic left heart syndrome in the United States, 1988–1997. Pediatrics. 2002;110(2 Pt 1):292–8. 12165581 10.1542/peds.110.2.292

[44] Checchia PA, McCollegan J, Daher N, Kolovos N, Levy F, Markovitz B. The effect of surgical case volume on outcome after the Norwood procedure. J Thorac Cardiovasc Surg. 2005;129(4):754–9. 15821640 10.1016/j.jtcvs.2004.07.056

[45] Gutgesell HP, Gibson J. Management of hypoplastic left heart syndrome in the 1990s. Am J Cardiol. 2002;89(7):842–6. 11909571 10.1016/s0002-9149(02)02196-3

[46] Hirsch JC, Gurney JG, Donohue JE, Gebremariam A, Bove EL, Ohye RG. Hospital mortality for Norwood and arterial switch operations as a function of institutional volume. Pediatr Cardiol. 2008;29(4):713–7. 18080151 10.1007/s00246-007-9171-2

[47] Hornik CP, He X, Jacobs JP, Li JS, Jaquiss RD, Jacobs ML, et al. Relative impact of surgeon and center volume on early mortality after the Norwood operation. Ann Thorac Surg. 2012;93(6):1992–7. 22516833 10.1016/j.athoracsur.2012.01.107 PMC3469698

[48] Karamlou T, McCrindle BW, Blackstone EH, Cai S, Jonas RA, Bradley SM, et al. Lesion-specific outcomes in neonates undergoing congenital heart surgery are related predominantly to patient and management factors rather than institution or surgeon experience: A Congenital Heart Surgeons Society Study. J Thorac Cardiovasc Surg. 2010;139(3):569–e771. 19909989 10.1016/j.jtcvs.2008.11.073

[49] McHugh KE, Hillman DG, Gurka MJ, Gutgesell HP. Three-stage palliation of hypoplastic left heart syndrome in the University HealthSystem Consortium. Congenit Heart Dis. 2010;5(1):8–15. 20136852 10.1111/j.1747-0803.2009.00367.x

[50] Tabbutt S, Ghanayem N, Ravishankar C, Sleeper LA, Cooper DS, Frank DU, et al. Risk factors for hospital morbidity and mortality after the Norwood procedure: A report from the Pediatric Heart Network Single Ventricle Reconstruction trial. J Thorac Cardiovasc Surg. 2012;144(4):882–95. 22704284 10.1016/j.jtcvs.2012.05.019 PMC4385520

[51] Welke KF, O’Brien SM, Peterson ED, Ungerleider RM, Jacobs ML, Jacobs JP. The complex relationship between pediatric cardiac surgical case volumes and mortality rates in a national clinical database. J Thorac Cardiovasc Surg. 2009;137(5):1133–40. 19379979 10.1016/j.jtcvs.2008.12.012

[52] Hunger R, Seliger B, Ogino S, Mantke R. Mortality factors in pancreatic surgery: A systematic review. How important is the hospital volume? Int J Surg. 2022;101:106640. 35525416 10.1016/j.ijsu.2022.106640 PMC9239346

[53] McAteer JP, LaRiviere CA, Drugas GT, Abdullah F, Oldham KT, Goldin AB. Influence of surgeon experience, hospital volume, and specialty designation on outcomes in pediatric surgery: a systematic review. JAMA Pediatr. 2013;167(5):468–75. 23529612 10.1001/jamapediatrics.2013.25

[54] Sakai-Bizmark R, Mena LA, Kumamaru H, Kawachi I, Marr EH, Webber EJ, et al. Impact of pediatric cardiac surgery regionalization on health care utilization and mortality. Health Serv Res. 2019;54(4):890–901. 30916392 10.1111/1475-6773.13137 PMC6606551

[55] Kansy A, Zu Eulenburg C, Sarris G, Jacobs JP, Fragata J, Tobota Z, et al. Higher Programmatic Volume in Neonatal Heart Surgery Is Associated With Lower Early Mortality. Ann Thorac Surg. 2018;105(5):1436–40. 29242060 10.1016/j.athoracsur.2017.11.028

[56] Morche J, Mathes T, Jacobs A, Wessel L, Neugebauer EAM, Pieper D. Relationship between volume and outcome for gastroschisis: A systematic review. J Pediatr Surg. 2022;57(12):763–85. 35459541 10.1016/j.jpedsurg.2022.03.022

[57] Morche J, Mathes T, Jacobs A, Pietsch B, Wessel L, Gruber S, et al. Relationship between volume and outcome for surgery on congenital diaphragmatic hernia: A systematic review. J Pediatr Surg. 2020;55(12):2555–65. 32376012 10.1016/j.jpedsurg.2020.03.025

[58] Sulkowski JP, Cooper JN, Lopez JJ, Jadcherla Y, Cuenot A, Mattei P, et al. Morbidity and mortality in patients with esophageal atresia. Surgery. 2014;156(2):483–91. 24947650 10.1016/j.surg.2014.03.016 PMC4099299

[59] Lawrence AE, Minneci PC, Deans KJ, Kelley-Quon LI, Cooper JN. Relationships between hospital and surgeon operative volumes and outcomes of esophageal atresia/tracheoesophageal fistula repair. J Pediatr Surg. 2019;54(1):44–9. 30401496 10.1016/j.jpedsurg.2018.10.037

[60] Trinh VT, Davies JM, Berger MS. Surgery for primary supratentorial brain tumors in the United States, 2000–2009: effect of provider and hospital caseload on complication rates. J Neurosurg. 2015;122(2):280–96. 25397366 10.3171/2014.9.JNS131648

[61] Pasquali SK, Wallace AS, Gaynor JW, Jacobs ML, O’Brien SM, Hill KD, et al. Congenital Heart Surgery Case Mix Across North American Centers and Impact on Performance Assessment. Ann Thorac Surg. 2016;102(5):1580–7. 27457827 10.1016/j.athoracsur.2016.04.034 PMC5077629

[62] Elassal AA, Al-Radi OO, Debis RS, Zaher ZF, Abdelmohsen GA, Faden MS, et al. Neonatal congenital heart surgery: contemporary outcomes and risk profile. J Cardiothorac Surg. 2022;17(1):80. 35443734 10.1186/s13019-022-01830-w PMC9022284

[63] Kansy A, Ebels T, Schreiber C, Tobota Z, Maruszewski B. Association of center volume with outcomes: analysis of verified data of European Association for Cardio-Thoracic Surgery Congenital Database. Ann Thorac Surg. 2014;98(6):2159–64. 25443021 10.1016/j.athoracsur.2014.07.065

[64] Hannan EL, Racz M, Kavey RE, Quaegebeur JM, Williams R. Pediatric cardiac surgery: the effect of hospital and surgeon volume on in-hospital mortality. Pediatrics. 1998;101(6):963–9. 9606220 10.1542/peds.101.6.963

[65] Karamlou T, Jacobs ML, Pasquali S, He X, Hill K, O’Brien S, et al. Surgeon and center volume influence on outcomes after arterial switch operation: analysis of the STS Congenital Heart Surgery Database. Ann Thorac Surg. 2014;98(3):904–11. 25069686 10.1016/j.athoracsur.2014.04.093

[66] Pasquali SK. Transforming Data Into Information. World J Pediatr Congenit Heart Surg. 2016;7(2):178–9. 26957400 10.1177/2150135115627652

[67] Mathes T, Zhang Z, Pachanov A, Pieper D. Systematic reviews and meta-analyses that include registry-based studies: methodological challenges and areas for future research. J Clin Epidemiol. 2023;156:119–22. 36806731 10.1016/j.jclinepi.2023.02.014

[68] Hernán MA, Robins JM. Using Big Data to Emulate a Target Trial When a Randomized Trial Is Not Available. Am J Epidemiol. 2016;183(8):758–64. 26994063 10.1093/aje/kwv254 PMC4832051

[69] Madenci AL, Wanis KN, Cooper Z, Haneuse S, Subramanian SV, Hofman A, et al. Strengthening Health Services Research Using Target Trial Emulation: An Application to Volume-Outcomes Studies. Am J Epidemiol. 2021;190(11):2453–60. 34089045 10.1093/aje/kwab170 PMC8799904

[70] Hampton Gray W, Sorabella RA, Moellinger AB, Zaccagni H, Padilla LA, Santiago B, et al. Standardization of the Norwood Procedure Improves Outcomes in a Medium-Sized Volume Center. World J Pediatr Congenit Heart Surg. 2024;15(6):738–45. 38853679 10.1177/21501351241249112

[71] Ismail MF, Elmahrouk AF, Arafat AA, Hamouda TE, Alshaikh BA, Shihata MS, et al. Evolution of the Norwood operation outcomes in patients with late presentation. J Thorac Cardiovasc Surg. 2020;159(3):1040–8. 31924357 10.1016/j.jtcvs.2019.07.154

[72] Mascio CE, Irons ML, Ittenbach RF, Gaynor JW, Fuller SM, Kaplinski M, et al. Thirty years and 1663 consecutive Norwood procedures: Has survival plateaued? J Thorac Cardiovasc Surg. 2019;158(1):220–9. 31248509 10.1016/j.jtcvs.2018.12.117

[73] Beqaj H, Goldshtrom N, Linder A, Buratto E, Setton M, DiLorenzo M, et al. Valved Sano conduit improves immediate outcomes following Norwood operation compared with nonvalved Sano conduit. J Thorac Cardiovasc Surg. 2024;167(4):1404–13. 37666412 10.1016/j.jtcvs.2023.08.036

[74] Hummel K, Whittaker S, Sillett N, Basken A, Berghammer M, Chalela T, et al. Development of an international standard set of clinical and patient-reported outcomes for children and adults with congenital heart disease: a report from the International Consortium for Health Outcomes Measurement Congenital Heart Disease Working Group. Eur Heart J Qual Care Clin Outcomes. 2021;7(4):354–65. 33576374 10.1093/ehjqcco/qcab009

