# Supplementary material for: An updated systematic review of the impact of volume of surgery and specialization in Norwood procedure
Source: BMC Pediatr. 2026 Jun 24;26:588. doi: 10.1186/s12887-026-07179-6 (PMC13295233; doi:10.1186/s12887-026-07179-6)
Supplement: Supplementary file 1 — Supplementary Material 1. [file 12887_2026_7179_MOESM1_ESM.docx]

## An updated systematic review of the impact of volume of surgery and specialization in Norwood procedure

### Supplementary file 1: Search strategies

### PubMed

("norwood procedures"[Mesh] OR Norwood[tiab] OR "Hypoplastic Left Heart Syndrome/surgery"[Mesh] OR hlhs[tiab] OR "Hypoplastic Left Heart Syndrome"[tiab]) AND (Volume[tiab] OR size[tiab] OR level[tiab] OR type[tiab] OR workload[tiab] OR caseload[tiab] OR centrali*[tiab] OR decentral*[tiab] OR regionali*[tiab] OR speciali*[tiab] OR "Hospitals, High-Volume"[Mesh] OR "Hospitals, Low-Volume"[Mesh] OR "Centralized Hospital Services"[Mesh] OR "Hospitals, Teaching"[Mesh] OR "Hospitals, Urban"[Mesh] OR "Hospitals, Rural"[Mesh]) AND ("Mortality"[Mesh] OR "Survival"[Mesh] OR "Disease-Free Survival"[Mesh] OR "Postoperative Complications"[Mesh] OR "Treatment Outcome"[Mesh] OR mortality[tiab] OR survival[tiab] OR complication*[tiab] OR outcom*[tiab] OR "Outcome and Process Assessment, Health Care"[Mesh]) AND (("2013/03/01"[Date - Publication] : "3000"[Date - Publication]))

Search on 17.01.2023
Hits: 712 results

Update on 30.12.2024
Hits: 158

### Embase

('norwood procedure'/exp OR norwood:ti,ab,kw OR 'hypoplastic left heart syndrome'/exp OR hlhs:ti,ab,kw OR 'hypoplastic left heart syndrome':ti,ab,kw) AND (volume:ti,ab,kw OR size:ti,ab,kw OR level:ti,ab,kw OR type:ti,ab,kw OR workload:ti,ab,kw OR caseload:ti,ab,kw OR centrali*:ti,ab,kw OR decentral*:ti,ab,kw OR regionali*:ti,ab,kw OR speciali*:ti,ab,kw OR 'high volume hospital'/exp OR 'high-volume surgeon'/exp OR 'low volume hospital'/exp OR 'low volume surgeon'/exp OR 'hospital management'/exp OR 'teaching hospital'/exp OR 'urban hospital'/exp OR 'rural hospital'/exp) AND ('mortality'/exp OR 'survival'/exp OR 'disease free survival'/exp OR 'postoperative complication'/exp OR 'treatment outcome'/exp OR mortality:ti,ab OR survival:ti,ab,kw OR complication*:ti,ab,kw OR outcom*:ti,ab,kw OR 'outcome assessment'/exp) AND [01-03-2013]/sd NOT [18-01-2023]/sd AND [embase]/lim NOT [medline]/lim NOT [preprint]/lim NOT [pubmed-not-medline]/lim

Search on 17.01.2023
Hits: 678

Update on 30.12.2024
Hits: 96

### Cochrane Library

#1        (Norwood OR hlhs OR “Hypoplastic Left Heart Syndrome”):ti,ab,kw

#2        [mh "Norwood Procedures"] OR [mh "Hypoplastic Left Heart Syndrome"/SU]

#3        #1 OR #2

#4        (Volume OR size OR level OR type OR workload OR caseload OR centrali* OR decentral* OR regionali* OR speciali*):ti,ab,kw

#5        [mh "Hospitals, High-Volume"] OR [mh "Hospitals, Low-Volume"] OR [mh "Centralized Hospital Services"] OR [mh "Hospitals, Teaching"] OR [mh "Hospitals, Urban"] OR [mh "Hospitals, Rural"]

#6        #4 OR #5

#7        [mh “Mortality”] OR [mh “Survival”] OR [mh "Disease-Free Survival"] OR [mh “Postoperative Complications”] OR [mh “Treatment Outcome”] OR [mh “Outcome and Process Assessment, Health Care”]

#8        (mortality OR survival OR complication* OR outcom*):ti,ab,kw

#9        #7 OR #8

#10       #3 AND #6 AND #9

with Cochrane Library publication date from Mar 2013 to Jan 2023

Search on 17.01.2023
Hits: 133

Update on 30.12.2024
Hits: 31
