# Supplementary material for: An updated systematic review of the impact of volume of surgery and specialization in Norwood procedure
Source: BMC Pediatr. 2026 Jun 24;26:588. doi: 10.1186/s12887-026-07179-6 (PMC13295233; doi:10.1186/s12887-026-07179-6)
Supplement: Supplementary file 2 — Supplementary Material 2. [file 12887_2026_7179_MOESM2_ESM.docx]

# An updated systematic review of the impact of volume of surgery and specialization in Norwood procedure

Supplementary file 2: Reports excluded during full-text screening

| **Reason for exclusion** | **Reference** |
| --- | --- |
| Conference abstract | Chamberlain, R. C., Andersen, N. D., McCrary, A. W., Hornik, C. P., & Hill, K. D. (2020). Acute Post-operative Renal Failure and Mortality Risk Following Norwood Surgical Palliation: A Secondary Analysis of the Pediatric Heart Network Single Ventricle Reconstruction Trial Public Use Database. *Circulation*, *142*(Suppl_3), A13533-A13533. |
|  | Foote, H., Thibault, D., Dominguez Gonzalez, C., Hill, G. D., Minich, L., McCrary, A., & Hill, K. D. (2022). Center Level Factors Associated With Improved Length of Stay Following Stage 1 Palliation: An Analysis of the NPC-QIC Registry. *Circulation*, *146*(Suppl_1), A10294-A10294. |
|  | Menon, S., McFadden, M., Wilkes, J., & Minich, L. L. (2013). Resource Utilization and Outcomes for Infants With Hypoplastic Left Heart Syndrome vs. Tricuspid Atresia: A Multi-Institutional Study. *Circulation*, *128*(Suppl_22), A12164-A12164 |
|  | Ta, H., Evers, P., Heydarian, H., Anderson, J., Brown, D., Marcuccio, E., ... & Hill, G. (2021). FREQUENCY AND FACTORS ASSOCIATED WITH AN IN-PATIENT INTERSTAGE: A REPORT FROM THE NPC-QIC. *Journal of the American College of Cardiology*, *77*(18_Supplement_1), 490-490. |
|  | Tunuguntla, H. P., Nembhard, W., Ethen, M. K., Canfield, M. A., Fixler, D. E., & Morris, S. A. (2013). The Impact of Institutional Stage I Surgical Volume on Mortality, Length of Stay, and Hospital Charges in Infants With Hypoplastic Left Heart Syndrome, a Review of the Texas Inpatient Public Use Data File From 1999-2012. *Circulation*, *128*(Suppl_22), A18001-A18001 |
|  | Zmora, R., Knight, J., Thomas, A. S., Bass, J., St Louis, J., Spector, L., & Kochilas, L. (2021). The Center-Volume Effect on Congenital Heart Surgery Revised: Is It Time to Consider Regionalization of Care?. *Circulation*, *144*(Suppl_1), A13689-A13689. |
|  | Nguyen M, Kops S, Kaizer A, Gilbert L, Londono-Obregon C, Villavicencio K, et al. Trends in Pulmonary Vein Doppler and Size Throughout Gestation in Fetuses with Hypoplastic Left Heart Syndrome. *Pediatric Cardiol*ogy, 44, S2-S3. |
| Wrong population | Delany, D. R., Chowdhury, S. M., Corrigan, C., & Buckley, J. R. (2022). Preoperative in-hospital mortality in neonates with critical CHD. *Cardiology in the Young*, *32*(11), 1794-1800. |
|  | Gupta, P., Jacobs, J. P., Pasquali, S. K., Hill, K. D., Gaynor, J. W., O’Brien, S. M., ... & Jacobs, M. L. (2014). Epidemiology and outcomes after in-hospital cardiac arrest after pediatric cardiac surgery. *The Annals of thoracic surgery*, *98*(6), 2138-2144. |
|  | Morris, S. A., Ethen, M. K., Penny, D. J., Canfield, M. A., Minard, C. G., Fixler, D. E., & Nembhard, W. N. (2014). Prenatal diagnosis, birth location, surgical center, and neonatal mortality in infants with hypoplastic left heart syndrome. *Circulation*, *129*(3), 285-292. |
| Wrong exposure | Hill, G. D., Bingler, M., McCoy, A. B., Oster, M. E., Uzark, K., & Bates, K. E. (2020). Improved national outcomes achieved in a cardiac learning health collaborative based on early performance level. *The Journal of pediatrics*, *222*, 186-192. |
|  | Newburger, J. W., Sleeper, L. A., Bellinger, D. C., Goldberg, C. S., Tabbutt, S., Lu, M., ... & Gaynor, J. W. (2012). Early developmental outcome in children with hypoplastic left heart syndrome and related anomalies: the single ventricle reconstruction trial. *Circulation*, *125*(17), 2081-2091. |
|  | Selenius, S., Ilvesvuo, J., Ruotsalainen, H., Mattila, I., Pätilä, T., Helle, E., & Ojala, T. (2023). Risk factors for mortality in patients with hypoplastic left heart syndrome after the Norwood procedure. *Interdisciplinary CardioVascular and Thoracic Surgery*, *37*(2) |
| Duplicate | Brown, K. L., Huang, Q., Hadjicosta, E., Seale, A. N., Tsang, V., Anderson, D., ... & Ridout, D. (2023). Long-term survival and center volume for functionally single-ventricle congenital heart disease in England and Wales. *The Journal of Thoracic and Cardiovascular Surgery*, *166*(2), 306-316. |
