# Supplementary material for: An updated systematic review of the impact of volume of surgery and specialization in Norwood procedure
Source: BMC Pediatr. 2026 Jun 24;26:588. doi: 10.1186/s12887-026-07179-6 (PMC13295233; doi:10.1186/s12887-026-07179-6)
Supplement: Supplementary file 3 — Supplementary Material 3. [file 12887_2026_7179_MOESM3_ESM.docx]

Table 1. Risk of bias assessment of studies on relationship between hospital, surgeon volume, hospital type and mortality-related outcomes

| **a. Risk of bias assessment of studies on relationship between hospital volume and mortality-related outcomes** | | | | | | | | | | |
| --- | --- | --- | --- | --- | --- | --- | --- | --- | --- | --- |
| **Study** | **Outcome** | **1. Risk of bias due to confounding** | **2. Risk of bias arising from measurement of the exposure** | **3. Risk of bias in selection of participants into the study (or into the analysis)** | **4. Risk of bias due to post-exposure interventions** | **5. Risk of bias due to missing data** | **6. Risk of bias arising from measurement of the outcome** | **7. Risk of bias in selection of the reported result** | **Overall risk of bias** |  |
| Anderson 2016 (1) | *In-hospital mortality^1^ | Low | Low | Low | Some concerns | Low | Low | Low | Some concerns |  |
| Anderson 2016 (1) | In-hospital mortality^2^ | Low | Low | Low | Some concerns | Low | Low | Low | Some concerns |  |
| Berry 2006 (2) | In-hospital mortality | Low | Low | Low | Some concerns | Low | Low | Low | Some concerns |  |
| Brown 2022 (3) | Long-term survival | Low | Low | Low | Some concerns | Low | Low | Low | Some concerns |  |
| Chang 2002 (4) | In-hospital mortality | NA | NA | NA | NA | NA | NA | NA | Very high risk |  |
| Chauhan 2024 (5) | *In-hospital mortality | High | Low | Low | High | Low | low | Low | High risk |  |
| Checchia 2005 (6) | In-hospital mortality | NA | NA | NA | NA | NA | NA | NA | Very high risk |  |
| Checchia 2005 (6) | 28-day survival | NA | NA | NA | NA | NA | NA | NA | Very high risk |  |
| Checchia 2005 (6) | Time to death | NA | NA | NA | NA | NA | NA | NA | Very high risk |  |
| Gong 2020 (7) | In-hospital mortality | High | low | Low | High | Low | low | Low | High risk |  |
| Gutgesell 2002 (8) | In-hospital mortality | NA | NA | NA | NA | NA | NA | NA | Very high risk |  |
| Hirsch 2008 (9) | *In-hospital mortality | High | Low | Low | Some concerns | Low | Low | low | High risk |  |
| Hornik 2012 (10)/ Pasquali 2012 (11) | *In-hospital mortality^3^ | Low | Low | Low | Some concerns | Low | Low | Low | Some concerns |  |
| Hornik 2012 (10)/ Pasquali 2012 (11) | In-hospital mortality^4^ | Low | Low | Low | Some concerns | Low | Low | Low | Some concerns |  |
| Karamlou 2010 (12) | 5-year mortality | Low | Low | Low | Some concerns | Low | Low | Low | Some concerns |  |
| McHugh 2010 (13)/ Dean 2013 (14) | In-hospital mortality^5^ | Low | Low | Low | Some concerns | Low | Low | Low | Some concerns |  |
| McHugh 2010 (13)/ Dean 2013 (14) | *In-hospital mortality^6^ | Low | Low | Low | Some concerns | Low | Low | Low | Some concerns |  |
| Schäfer 2025 (15) | 1-year mortality | Low | Low | Low | Some concerns | High | Low | Low | High risk |  |
| Schäfer 2025 (15) | 30-day mortality | Low | Low | Low | Some concerns | High | Low | Low | High risk |  |
| Welke 2023 (16) | Operative mortality | Low | Low | Low | Some concerns | Low | Low | Low | Some concerns |  |
| Welke 2009 (17) | *In-hospital mortality | Low | Low | Low | Some concerns | Low | Low | Low | Some concerns |  |
| Yoshimura 2023 (18) | Operative mortality | Low | Low | Low | Some concerns | Low | Low | Low | Some concerns |  |
| Zmora 2023 (19) | In-hospital mortality | Low | Low | Low | Some concerns | Low | Low | Low | Some concerns |  |
| Zmora 2023 (19) | 90-day mortality | Low | Low | Low | Some concerns | Low | Low | Low | Some concerns |  |
| Zmora 2023 (19) | 1-year mortality | Low | Low | Low | Some concerns | Low | Low | Low | Some concerns |  |
| Zmora 2023 (19) | *1-year mortality (deaths within 90 d. excluded) | Low | Low | High | Some concerns | Low | Low | Low | High risk |  |
| Zmora 2023 (19) | 3-year mortality | Low | Low | Low | Some concerns | Low | Low | Low | Some concerns |  |
| Zmora 2023 (19) | *3-year mortality (deaths within 90 d. excluded) | Low | Low | High | Some concerns | Low | Low | Low | High risk |  |
| **b. Risk of bias assessment of studies on relationship between surgeon volume and mortality-related outcomes** | | | | | | | | | | |
| **Study** | **Outcome** | **1. Risk of bias due to confounding** | **2. Risk of bias arising from measurement of the exposure** | **3. Risk of bias in selection of participants into the study (or into the analysis)** | **4. Risk of bias due to post-exposure interventions** | **5. Risk of bias due to missing data** | **6. Risk of bias arising from measurement of the outcome** | **7. Risk of bias in selection of the reported result** | **Overall risk of bias** |  |
| Anderson 2016 (1) | *In-hospital mortality^1^ | Low | Low | Low | Some concerns | Low | Low | Low | Some concerns |  |
| Anderson 2016 (1) | In-hospital mortality^7^ | Low | Low | Low | Some concerns | Low | Low | Low | Some concerns |  |
| Chechia 2005 (6) | 28-day survival | NA | NA | NA | NA | NA | NA | NA | Very high risk |  |
| Chechia 2005 (6) | In-hospital mortality | NA | NA | NA | NA | NA | NA | NA | Very high risk |  |
| Hornik 2012 (10)/ Pasquali 2012 (11) | In-hospital mortality^3^ | Low | Low | Low | Some concerns | Low | Low | Low | Some concerns |  |
| Karamlou 2010 (12) | 5-year mortality | Low | Low | Low | Some concerns | Low | Low | Low | Some concerns |  |
| SVR Trial  (Jean-St-Michel 2018 (20)) | Death or transplantation before stage II | High | Low | Some concerns | Some concerns | Low | Low | Low | High risk |  |
| SVR Trial (Newburger 2014 (21)) | 3-year death or transplantation-free survival | Low | Low | Low | Some concerns | low | low | low | Some concerns |  |
| SVR Trial (Newburger 2018 (22)) | 6-year death or transplantation-free survival | Low | Low | Low | Some concerns | Low | Low | Low | Some concerns |  |
| **c. Risk of bias assessment of studies on relationship between hospital type and mortality-related outcomes** | | | | | | | | | | |
| **Study** | **Outcome** | **1. Risk of bias due to confounding** | **2. Risk of bias arising from measurement of the exposure** | **3. Risk of bias in selection of participants into the study (or into the analysis)** | **4. Risk of bias due to post-exposure interventions** | **5. Risk of bias due to missing data** | **6. Risk of bias arising from measurement of the outcome** | **7. Risk of bias in selection of the reported result** | **Overall risk of bias** |  |
| Berry 2006 (2) | In-hospital mortality | Low | Low | Low | Some concerns | Low | Low | Low | Some concerns |  |
| Hirsch 2008 (9) | In-hospital mortality | NA | NA | NA | NA | NA | NA | NA | Very high risk |  |

NA = not applicable (according to the ROBINS-E Preliminary Considerations B: studies with sufficient potential for confounding and no attempt to control for it should be considered at very high risk of bias, and no further assessment is required); SVR = single ventricle reconstruction
*Study/analysis excluded from final synthesis
^1^ Model adjusted for patient characteristics
^2^ Model adjusted for patient characteristics and surgeon volume
^3^ Analysis of Hornik et al. (10)
^4^ Analysis of Pasquali et al. (11)
^5^ Analysis of McHugh et al. (13)
^6^ Analysis of Dean et al. (14)
^7^ Model adjusted for patient characteristics and hospital volume

Table 2. Risk of bias assessment of studies on relationship between hospital, surgeon volume and secondary outcomes

| **a. Risk of bias assessment of studies on relationship between hospital volume and secondary outcomes** | | | | | | | | | | |
| --- | --- | --- | --- | --- | --- | --- | --- | --- | --- | --- |
| **Study** | **Outcome** | **1. Risk of bias due to confounding** | **2. Risk of bias arising from measurement of the exposure** | **3. Risk of bias in selection of participants into the study (or into the analysis)** | **4. Risk of bias due to post-exposure interventions** | **5. Risk of bias due to missing data** | **6. Risk of bias arising from measurement of the outcome** | **7. Risk of bias in selection of the reported result** | **Overall risk of bias** |  |
| Anderson 2016 (1) | LOS overall | NA | NA | NA | NA | NA | NA | NA | Very high risk |  |
| Anderson 2016 (1) | *LOS for survivors | NA | NA | NA | NA | NA | NA | NA | Very high risk |  |
| Anderson 2016 (1) | *Postoperative LOS (uncensored)^1^ | Low | Low | Low | Some concerns | Low | Low | Some concerns | Some concerns |  |
| Anderson 2016 (1) | *Postoperative LOS (uncensored)^2^ | Low | Low | Low | Some concerns | Low | Low | Some concerns | Some concerns |  |
| Anderson 2016 (1) | *Postoperative LOS (censored)^1^ | Low | Low | Low | Some concerns | Low | Low | Some concerns | Some concerns |  |
| Anderson 2016 (1) | *Postoperative LOS (censored)^2^ | Low | Low | Low | Some concerns | Low | Low | Some concerns | Some concerns |  |
| SVR Trial (Chamberlain 2022 (23)) | *Renal failure | Low | Low | Low | Some concerns | Low | low | low | Some concerns |  |
| Checchia 2005 (6) | Mean LOS in survivors | NA | NA | NA | NA | NA | NA | NA | Very high risk |  |
| Gong 2020 (7) | LOS overall | High | Low | Low | High | Low | Low | Low | High risk |  |
| Gong 2020 (7) | *LOS, stratified, ECMO | High | Low | Low | High | Low | Low | Low | High risk |  |
| Gong 2020 (7) | *LOS, stratified, non-ECMO | High | Low | Low | High | Low | Low | Low | High risk |  |
| Gong 2020 (7) | *LOS, stratified, survivors | High | Low | High | High | Low | Low | Low | High risk |  |
| Gong 2020 (7) | *LOS, stratified, non-survivors | High | Low | High | High | Low | Low | Low | High risk |  |
| Gong 2020 (7) | ECMO use | Low | Low | Low | Some concerns | Low | Low | Low | Some concerns |  |
| Schäfer 2025 (15) | Need for unplanned catheterization | Low | Low | Low | Some concerns | High | Low | Low | High risk |  |
| SVR Trial  (Tabbutt 2012 (24)) | Log length of ventilation in days | High | Low | Low | Some concerns | Low | Low | Low | High risk |  |
| SVR Trial  (Tabbutt 2012 (24)) | Log LOS | High | Low | Low | Some concerns | Low | Low | Low | High risk |  |
| SVR Trial (Tabbutt 2012 (24)) | Log time to first extubation | High | Low | Low | Some concerns | Low | Low | Low | High risk |  |
| SVR Trial  (Tabbutt 2012 (24)) | Renal failure | Low | Low | Low | Some concerns | Low | Low | Low | Some concerns |  |
| SVR Trial  (Tabbutt 2012 (24)) | Sepsis | Low | Low | Low | Some concerns | Low | Low | Low | Some concerns |  |
| Welke 2023 (16) | Major complications | Low | Low | Low | Some concerns | Low | Low | Low | Some concerns |  |
| Welke 2023 (16) | FTR | Low | Low | Low | Some concerns | Low | Low | Low | Some concerns |  |
| Welke 2023 (16) | LOS overall | Low | Low | Low | Some concerns | Low | Low | Low | Some concerns |  |
| Welke 2023 (16) | *LOS among survivors | Low | Low | High | Some concerns | Low | Low | Low | High risk |  |
| **b. Risk of bias assessment of studies on relationship between surgeon volume and secondary outcomes** | | | | | | | | | | |
| **Study** | **Outcome** | **1. Risk of bias due to confounding** | **2. Risk of bias arising from measurement of the exposure** | **3. Risk of bias in selection of participants into the study (or into the analysis)** | **4. Risk of bias due to post-exposure interventions** | **5. Risk of bias due to missing data** | **6. Risk of bias arising from measurement of the outcome** | **7. Risk of bias in selection of the reported result** | **Overall risk of bias** |  |
| Anderson 2016 (1) | LOS | NA | NA | NA | NA | NA | NA | NA | Very high risk |  |
| Anderson 2016 (1) | *LOS for survivors | NA | NA | NA | NA | NA | NA | NA | Very high risk |  |
| Anderson 2016 (1) | *Postoperative LOS (censored)^1^ | Low | Low | Low | Some concerns | Low | Low | Some concerns | Some concerns |  |
| Anderson 2016 (1) | *Postoperative LOS (censored)^3^ | Low | Low | Low | Some concerns | Low | Low | Some concerns | Some concerns |  |
| Anderson 2016 (1) | *Postoperative LOS (uncensored)^1^ | Low | Low | Low | Some concerns | Low | Low | Some concerns | Some concerns |  |
| Anderson 2016 (1) | *Postoperative LOS (uncensored)^3^ | Low | Low | Low | Some concerns | Low | Low | Some concerns | Some concerns |  |
| SVR Trial  (Tabbutt 2012 (24)) | Renal failure | Low | Low | Low | Some concerns | Low | Low | Low | Some concerns |  |
| SVR Trial  (Tabbutt 2012 (24)) | Log time to first extubation | High | Low | Low | Some concerns | Low | Low | Low | High risk |  |
| SVR Trial  (Tabbutt 2012 (24)) | Log length of ventilation in days | High | Low | Low | Some concerns | Low | Low | Low | High risk |  |

NA = not applicable (according to the ROBINS-E Preliminary Considerations B: studies with sufficient potential for confounding and no attempt to control for it should be considered at very high risk of bias, and no further assessment is required); SVR = single ventricle reconstruction
*Study/analysis excluded from final synthesis
^1^ Model adjusted for patient characteristics
^2^ Model adjusted for patient characteristics and surgeon volume
^3^ Model adjusted for patient characteristics and hospital volume

## References

1. Anderson BR, Ciarleglio AJ, Cohen DJ, Lai WW, Neidell M, Hall M, et al. The Norwood operation: Relative effects of surgeon and institutional volumes on outcomes and resource utilization. Cardiol Young. 2016;26(4):683-92.

2. Berry JG, Cowley CG, Hoff CJ, Srivastava R. In-hospital mortality for children with hypoplastic left heart syndrome after stage I surgical palliation: teaching versus nonteaching hospitals. Pediatrics. 2006;117(4):1307-13.

3. Brown KL, Huang Q, Hadjicosta E, Seale AN, Tsang V, Anderson D, et al. Long-term survival and center volume for functionally single-ventricle congenital heart disease in England and Wales. J Thorac Cardiovasc Surg. 2022.

4. Chang RK, Chen AY, Klitzner TS. Clinical management of infants with hypoplastic left heart syndrome in the United States, 1988-1997. Pediatrics. 2002;110(2 Pt 1):292-8.

5. Chauhan D, Mehaffey JH, Hayanga JWA, Udassi JP, Badhwar V, Mascio CE. Volume Alone Does Not Predict Quality Outcomes in Hospitals Performing Pediatric Cardiac Surgery. Ann Thorac Surg. 2024;117(6):1187-93.

6. Checchia PA, McCollegan J, Daher N, Kolovos N, Levy F, Markovitz B. The effect of surgical case volume on outcome after the Norwood procedure. J Thorac Cardiovasc Surg. 2005;129(4):754-9.

7. Gong CL, Song AY, Horak R, Friedlich PS, Lakshmanan A, Pruetz JD, et al. Impact of Confounding on Cost, Survival, and Length-of-Stay Outcomes for Neonates with Hypoplastic Left Heart Syndrome Undergoing Stage 1 Palliation Surgery. Pediatr Cardiol. 2020;41(5):996-1011.

8. Gutgesell HP, Gibson J. Management of hypoplastic left heart syndrome in the 1990s. Am J Cardiol. 2002;89(7):842-6.

9. Hirsch JC, Gurney JG, Donohue JE, Gebremariam A, Bove EL, Ohye RG. Hospital mortality for Norwood and arterial switch operations as a function of institutional volume. Pediatr Cardiol. 2008;29(4):713-7.

10. Hornik CP, He X, Jacobs JP, Li JS, Jaquiss RD, Jacobs ML, et al. Relative impact of surgeon and center volume on early mortality after the Norwood operation. Ann Thorac Surg. 2012;93(6):1992-7.

11. Pasquali SK, Jacobs JP, He X, Hornik CP, Jaquiss RD, Jacobs ML, et al. The complex relationship between center volume and outcome in patients undergoing the Norwood operation. Ann Thorac Surg. 2012;93(5):1556-62.

12. Karamlou T, McCrindle BW, Blackstone EH, Cai S, Jonas RA, Bradley SM, et al. Lesion-specific outcomes in neonates undergoing congenital heart surgery are related predominantly to patient and management factors rather than institution or surgeon experience: A Congenital Heart Surgeons Society Study. J Thorac Cardiovasc Surg. 2010;139(3):569-77.e1.

13. McHugh KE, Hillman DG, Gurka MJ, Gutgesell HP. Three-stage palliation of hypoplastic left heart syndrome in the University HealthSystem Consortium. Congenit Heart Dis. 2010;5(1):8-15.

14. Dean PN, McHugh K, Hillman DG, Conaway MR, Gutgesell H. Effects of race, ethnicity and gender on surgical mortality for hypoplastic left heart syndrome. Journal of the American College of Cardiology. 2013;61(10):E431.

15. Schäfer M, McFarland C, Amula V, Truong D, Lambert LM, Griffiths ER, et al. Volume-Outcome Relationship of Norwood Procedures: Insights from the National Pediatric Cardiology-Quality Improvement Collaborative Database. Ann Thorac Surg. 2025;119(5):1045-52.

16. Welke KF, Karamlou T, O'Brien SM, Dearani JA, Tweddell JS, Kumar SR, et al. Contemporary Relationship Between Hospital Volume and Outcomes in Congenital Heart Surgery. Ann Thorac Surg. 2023;116(6):1233-9.

17. Welke KF, O'Brien SM, Peterson ED, Ungerleider RM, Jacobs ML, Jacobs JP. The complex relationship between pediatric cardiac surgical case volumes and mortality rates in a national clinical database. J Thorac Cardiovasc Surg. 2009;137(5):1133-40.

18. Yoshimura N, Hirata Y, Inuzuka R, Tachimori H, Hirano A, Sakurai T, et al. Effect of procedural volume on the outcomes of congenital heart surgery in Japan. J Thorac Cardiovasc Surg. 2023;165(4):1541-50.e3.

19. Zmora R, Spector L, Bass J, Thomas A, Knight J, Lakshminarayan K, et al. Procedure-Specific Center Volume and Mortality After Infantile Congenital Heart Surgery. Ann Thorac Surg. 2023;116(3):525-31.

20. Jean-St-Michel E, Meza JM, Maguire J, Coles J, McCrindle BW. Survival to Stage II with Ventricular Dysfunction: Secondary Analysis of the Single Ventricle Reconstruction Trial. Pediatr Cardiol. 2018;39(5):955-66.

21. Newburger JW, Sleeper LA, Frommelt PC, Pearson GD, Mahle WT, Chen S, et al. Transplantation-free survival and interventions at 3 years in the single ventricle reconstruction trial. Circulation. 2014;129(20):2013-20.

22. Newburger JW, Sleeper LA, Gaynor JW, Hollenbeck-Pringle D, Frommelt PC, Li JS, et al. Transplant-Free Survival and Interventions at 6 Years in the SVR Trial. Circulation. 2018;137(21):2246-53.

23. Chamberlain RC, Andersen ND, McCrary AW, Hornik CP, Hill KD. Postoperative Renal Failure, Shunt Type, and Mortality After Norwood Palliation. Ann Thorac Surg. 2022;113(6):2046-53.

24. Tabbutt S, Ghanayem N, Ravishankar C, Sleeper LA, Cooper DS, Frank DU, et al. Risk factors for hospital morbidity and mortality after the Norwood procedure: A report from the Pediatric Heart Network Single Ventricle Reconstruction trial. J Thorac Cardiovasc Surg. 2012;144(4):882-95.
