# Supplementary material for: An updated systematic review of the impact of volume of surgery and specialization in Norwood procedure
Source: BMC Pediatr. 2026 Jun 24;26:588. doi: 10.1186/s12887-026-07179-6 (PMC13295233; doi:10.1186/s12887-026-07179-6)
Supplement: Supplementary file 5 — Supplementary Material 5. [file 12887_2026_7179_MOESM5_ESM.docx]

# An updated systematic review of the impact of volume of surgery and specialization in Norwood procedure

## Supplementary file 5: Analyses excluded from the final synthesis

Table 1. Analyses of mortality-related outcomes excluded from final synthesis

| **Table 1a. Analyses on association between hospital volume and mortality-related outcomes** | | | | | |
| --- | --- | --- | --- | --- | --- |
| **Study ID** | **Outcome** | **Analysis/Model  (adjustment)** | **Volume** | **Effect measure** | **Overall risk of bias** |
| Anderson 2016 (1) | In-hospital mortality | Logistic regression (patient characteristics: sex, birth weight, prematurity, major co-morbid condition, dominant right ventricle, year of admission, long pre-operative time, and insurance type) | as a continuous variable:  with units of 10 operations/year | OR 0.82 (CI: 0.74-0.90) | Some concerns |
| Checchia 2005 (2) | Time to death | No model | <16/4 years 16-30/4 years >30/4 years | Mean (SD) 19.6 (± 33.2) 12.2 (± 9.7) 20.2 (± 9.6) p > 0.05 | Very high risk |
| Hornik 2012 (3)/ Pasquali 2012 (4) | In-hospital mortality | Hornik 2012/ Logistic regression (year of surgery, age, weight, sex, dominant ventricle, diagnosis of total anomalous pulmonary venous return, preoperative length of stay, the presence of any noncardiac or genetic abnormality and preoperative shock, mechanical ventilatory or circulatory support, arrhythmia, neurologic deficit, surgeon volume) | ≤10 11-20 ≥20 | OR 1.37 (CI: 0.92-2.05) OR 1.20 (CI: 0.80-1.82) Ref. | Some concerns |
| McHugh 2010 (5)/ Dean 2013 (6) | In-hospital mortality | Dean 2013/ Logistic regression (gender, race, surgical era, admission category) | >124/10 years ≤124/10 years | OR 0.57 (CI: 0.45-0.71) Ref. | Some concerns |
| Zmora 2023 (7) | 1-year mortality | Deaths within 90 d. excluded/ Logistic regression model (age (days) and weight z-score at the time of surgery, sex, chromosomal abnormality, and surgical era) | as a continuous variable: 1-unit increase/year | OR 1.02 (CI: 0.98-1.06) | High risk |
| Zmora 2023 (7) | 3-year mortality | Deaths within 90 d. excluded/ Logistic regression model (age (days) and weight z-score at the time of surgery, sex, chromosomal abnormality, and surgical era) | as a continuous variable: 1-unit increase/year | OR 1.01 (CI: 0.99-1.03) | High risk |
| **Table 1b. Analysis on association between surgeon volume and mortality-related outcomes** | | | | | |

| **Study ID** | **Outcome** | **Analysis/Model  (adjustment)** | **Volume** | **Effect measure** | **Overall risk of bias** |
| --- | --- | --- | --- | --- | --- |
| Anderson 2016 (1) | In-hospital mortality | Logistic regression (patient characteristics) | as a continuous variable:  with units of 5 operations/year | OR 1.02 (95% CI: 0.84-1.24) | Some concerns |

| OR=odds ratio; CI=95% confidence interval; SD=standard deviation; Ref.=reference |
| --- |

Table 2. Analyses of secondary outcomes excluded from final synthesis

| **Table 2a. Analyses on association between hospital volume and secondary outcomes** | | | | | |
| --- | --- | --- | --- | --- | --- |
| **Study ID** | **Outcome** | **Analysis/Model (adjustment)** | **Volume** | **Effect measure** | **Overall risk of bias** |
| Anderson 2016 (1) | LOS for survivors | No model | ≤10/year 10-19/year >19/year | 25 (IQR: 15-46)  24 (IQR: 15-40) 22 (IQR: 13-39) p-value=0.005 | Very high risk |
|  | Postoperative LOS  (censored) | Linear regression (patient characteristics) | as a continuous variable: with units of 10 operations per year | 1. eβ 0.92 (CI: 0.86-0.98) | Some concerns |
|  | Postoperative LOS  (censored) | Linear regression (patient characteristics and surgeon volume) | as a continuous variable: with units of 10 operations per year | eβ 0.89 (CI: 0.82-0.96) | Some concerns |
|  | Postoperative LOS (uncensored) | Linear regression (patient characteristics) | as a continuous variable: with units of 10 operations per year | eβ 0.95 (CI: 0.90-1.00) | Some concerns |
|  | Postoperative LOS (uncensored) | Linear regression (patient characteristics and surgeon volume) | as a continuous variable: with units of 10 operations per year | eβ 0.93 (CI: 0.86-1.00) | Some concerns |
| Gong  2020 (8) | LOS, stratified, ECMO | Linear regression (gender; Race/ethnicity; payer type; hospital region; income; No. of complex chronic conditions; comorbidities; Mortality) | <11/year 11-25/year >25/year | Ref. 17.09 (CI: 0.82-33.37; p=0.04) 2.96 (CI: -16.13-22.05; p=0.76) | High risk |
|  | LOS, stratified, non-ECMO | Linear regression (gender; Race/ethnicity; payer type; hospital region; income; No. of complex chronic conditions; comorbidities; Mortality) | <11/year 11-25/year >25/year | Ref. 1.05 (CI: -4.23-6.33; p=0.70) 2.93 (CI: -3.05-8.92; p=0.34) | High risk |
|  | LOS, stratified, non-survivors | Linear regression (gender; Race/ethnicity; payer type; hospital region; income; No. of complex chronic conditions; comorbidities; ECMO) | <11/year 11-25/year >25/year | Ref. 20.64 (CI: 4.31-36.97; p=0.01) 7.44 (CI: -11.21-26.09; p=0.43) | High risk |
|  | LOS, stratified, survivors | Linear regression (gender; Race/ethnicity; payer type; hospital region; income; No. of complex chronic conditions; comorbidities; ECMO) | <11/year 11-25/year >25/year | Ref. 0.05 (CI:-5.07-5.17; p=0.99) 1.86 (CI:-3.95-7.67; p=0.53) | High risk |
| Welke  2023 (9) | Postoperative LOS among survivors | Linear regression (prematurity, prior cardiovascular operation, shock, renal failure, preoperative ventilator support, any other preoperative risk factor, any noncardiac congenital anatomic abnormality, chromosomal abnormality/syndrome categories with categories 3 to 5 collapsed together, age, and weight) | as a continuous variable (congenital heart surgery):  50 vs 450/year  100 vs 450/year  200 vs 450/year  300 vs 450/year | OR 1.26 (CI: 1.04-1.51)  OR 1.17 (CI: 0.98-1.38)  OR 1.05 (CI: 0.86-1.26)  OR 1.00 (CI: 0.89-1.13) | High risk |
| **Table 2b. Analysis on association between surgeon volume and secondary outcomes** | | | | | |
| \| **Study ID** \| **Outcome** \| **Analysis/Model  (adjustment)** \| **Volume** \| **Effect measure** \| **Overall risk of bias** \| \| --- \| --- \| --- \| --- \| --- \| --- \| \| Anderson 2016 (1) \| LOS for survivors \| No model \| ≤10/year 10-19/year >19/year \| 23 (IQR 14-40) 26 (IQR 16-44) 23 (13-41)  p-value=0.423 \| Very high risk \| \| Postoperative LOS (censored) \| Linear regression (patient characteristics) \| as a continuous variable: with units of 5 operations per year \| eβ 0.99 (CI: 0.92-1.06) \| Some concerns \| \| Postoperative LOS (censored) \| Linear regression (patient characteristics and hospital volume) \| as a continuous variable: with units of 5 operations per year \| eβ 0.99 (CI: 0.93-1.05) \| Some concerns \| \| Postoperative LOS (uncensored) \| Linear regression (patient characteristics) \| as a continuous variable: with units of 5 operations per year \| eβ 0.99 (CI: 0.95-1.03) \| Some concerns \| \| Postoperative LOS (uncensored) \| Linear regressions (patient characteristics and hospital volume) \| as a continuous variable: with units of 5 operations per year \| eβ 0.99 (CI: 0.93-1.05) \| Some concerns \| | | | | | |
| LOS=length of stay; eβ=back-transformed coefficients; CI=95% confidence interval; IQR=interquartile range; ECMO=extracorporeal membrane oxygenation; Ref.=reference | | | | | |

# References

1. Anderson BR, Ciarleglio AJ, Cohen DJ, Lai WW, Neidell M, Hall M, et al. The Norwood operation: Relative effects of surgeon and institutional volumes on outcomes and resource utilization. Cardiol Young. 2016;26(4):683-92.

2. Checchia PA, McCollegan J, Daher N, Kolovos N, Levy F, Markovitz B. The effect of surgical case volume on outcome after the Norwood procedure. J Thorac Cardiovasc Surg. 2005;129(4):754-9.

3. Hornik CP, He X, Jacobs JP, Li JS, Jaquiss RD, Jacobs ML, et al. Relative impact of surgeon and center volume on early mortality after the Norwood operation. Ann Thorac Surg. 2012;93(6):1992-7.

4. Pasquali SK, Jacobs JP, He X, Hornik CP, Jaquiss RD, Jacobs ML, et al. The complex relationship between center volume and outcome in patients undergoing the Norwood operation. Ann Thorac Surg. 2012;93(5):1556-62.

5. McHugh KE, Hillman DG, Gurka MJ, Gutgesell HP. Three-stage palliation of hypoplastic left heart syndrome in the University HealthSystem Consortium. Congenit Heart Dis. 2010;5(1):8-15.

6. Dean PN, McHugh K, Hillman DG, Conaway MR, Gutgesell H. Effects of race, ethnicity and gender on surgical mortality for hypoplastic left heart syndrome. Journal of the American College of Cardiology. 2013;61(10):E431.

7. Zmora R, Spector L, Bass J, Thomas A, Knight J, Lakshminarayan K, et al. Procedure-Specific Center Volume and Mortality After Infantile Congenital Heart Surgery. Ann Thorac Surg. 2023;116(3):525-31.

8. Gong CL, Song AY, Horak R, Friedlich PS, Lakshmanan A, Pruetz JD, et al. Impact of Confounding on Cost, Survival, and Length-of-Stay Outcomes for Neonates with Hypoplastic Left Heart Syndrome Undergoing Stage 1 Palliation Surgery. Pediatr Cardiol. 2020;41(5):996-1011.

9. Welke KF, Karamlou T, O'Brien SM, Dearani JA, Tweddell JS, Kumar SR, et al. Contemporary Relationship Between Hospital Volume and Outcomes in Congenital Heart Surgery. Ann Thorac Surg. 2023;116(6):1233-9.
