# Supplementary material for: An updated systematic review of the impact of volume of surgery and specialization in Norwood procedure
Source: BMC Pediatr. 2026 Jun 24;26:588. doi: 10.1186/s12887-026-07179-6 (PMC13295233; doi:10.1186/s12887-026-07179-6)
Supplement: Supplementary file 6 — Supplementary Material 6. [file 12887_2026_7179_MOESM6_ESM.docx]

# An updated systematic review of the impact of volume of surgery and specialization in Norwood procedure

## Supplementary file 6: Analyses examining secondary outcomes

Table 1. Results of studies examining association between hospital volume and secondary outcomes

| **Study ID** | **Outcome** | **Analysis/Model (adjustment)** | **Volume** | **Effect measure** | **Overall risk of bias** |
| --- | --- | --- | --- | --- | --- |
| Anderson 2016 (1) | Median LOS overall | No model | ≤10/year 10-19/year >19/year | 32 (IQR: 21-54) 30 (IQR: 20-47) 27 (IQR: 17-45) p-value<0.001 | Very high risk |
| Checchia 2005 (2) | Mean LOS in survivors | Linear regression | <16/4 years 16-30/4 years >30/4 years | 36.5 (SD: ± 32.4) 28.7 (SD: ± 8.4) 29.4 (SD: ± 5.7) p > 0.05 | Very high risk |
| Gong  2020 (3) | ECMO use | Logistic regression (gender; Race/ethnicity; payer type; hospital region; income; No. of complex chronic conditions; comorbidities; LOS) | <11/year 11-25/year >27/year | Ref. OR 0.68 (CI: 0.51-0.90, E=1.72) OR 0.72 (CI: 0.52-1.01, E=1) | Some concerns |
| Gong  2020 (3) | LOS, overall | Linear regression (gender; Race/ethnicity; payer type; hospital region; income; No. of complex chronic conditions; comorbidities; ECMO; Mortality) | <11/year 11-25/year >25/year | Ref. 2.94 (CI: -2.19-8.08; p=0.26) 2.58 (CI: -3.25-8.42; p=0.39) | High risk |
| Schäfer  2025 (4) | Need for unplanned catheterization | Logistic regression (age, weight, sex, and mode of surgical intervention) | ≤5/year 6–10/year >10/year | OR 1.41 (CI: 1.13–1.75) OR 1.40 (CI: 1.19–1.65) Ref. | High risk |
| SVR Trial/ Tabbutt 2012 (5) | Log length of ventilation in days | Linear regression (gestational age, genetic abnormality, preoperative intubation, left atrial decompression, preoperative shock, TR preoperatively, age, open sternum, operations after Norwood procedure, surgeon volume) | ≤15/year 16 to 20/year  21 to 30/year  >30/year | 0.004 0.26 0.12 Ref. p=0.005 | High risk |
| SVR Trial/ Tabbutt 2012 (5) | Log LOS | Linear regression (birth weight, genetic abnormality, preoperative intubation for shock, TR preoperative, duration of DHCA, operations after Norwood procedure, surgeon volume) | ≤15/year 16 to 20/year  21 to 30/year  >30/year | 0.16 0.34 -0.03 Ref p<0.001 | High risk |
| SVR Trial/ Tabbutt 2012 (5) | Log time to first extubation | Linear regression (gestational age, left atrial decompression, TR preoperatively, duration of regional cerebral perfusion, ECMO, open sternum, duration of open sternum, operations after Norwood procedure, surgeon volume) | ≤15/year 16 to 20/year  21 to 30/year  >30/year | -0.06 0.31 0.21 Ref. p<0.001 | High risk |
| SVR Trial/ Tabbutt 2012 (5) | Sepsis | Logistic regression (gestational age, AS/MS/VD, duration of DHCA, open sternum duration) | ≤15/year 16 to 20/year  21 to 30/year  >30/year | OR 2.28 (CI: 1.17-4.47) OR 0.94 (CI: 0.40-2.19) OR 0.64 (CI: 0.33-1.26) Ref. p<0.001 | Some concerns |
| SVR Trial/ Tabbutt 2012 (5) | Renal failure | Logistic regression (anomalous pulmonary venous return, preoperative intubation, heart block, open sternum, volume) | ≤15/year 16 to 20/year  21 to 30/year  >30/year | OR 1.55 (CI: 0.53-4.58) OR 0.44 (CI: 0.14-1.45) OR 0.32 (CI: 0.11-0.91) Ref. p=0.02 | Some concerns |
| Welke  2023 (6) | Postoperative LOS overall | Linear regression (prematurity, prior cardiovascular operation, shock, renal failure, preoperative ventilator support, any other preoperative risk factor, any noncardiac congenital anatomic abnormality, chromosomal abnormality/syndrome categories with categories 3 to 5 collapsed together, age, and weight) | as a continuous variable (congenital heart surgery):  50 vs 450/year  100 vs 450/year  200 vs 450/year  300 vs 450/year | OR 1.01 (CrI: 0.83-1.21) OR 1.01 (CrI: 0.85-1.18) OR 1.01 (CrI: 0.84-1.20) OR 1.00 (CrI: 0.89-1.12) | Some concerns |
| Welke  2023 (6) | Major complications | Logistic regression model (prematurity, prior cardiovascular operation, shock, renal failure, preoperative ventilator support, any other preoperative risk factor, any noncardiac congenital anatomic abnormality, chromosomal abnormality/syndrome categories with categories 3 to 5 collapsed together, age, and weight) | as a continuous variable (congenital heart surgery): 50 vs 450/year 100 vs 450/year 200 vs 450/year 300 vs 450/year | OR 1.81 (CrI: 0.95-3.20) OR 1.58 (CrI: 0.88-2.65) OR 1.29 (CrI:0.69-2.27) OR 1.11 (95% CrI 0.75-1.60) | Some concerns |
| Welke  2023 (6) | Failure to rescue | Logistic regression model (prematurity, prior cardiovascular operation, shock, renal failure, preoperative ventilator support, any other preoperative risk factor, any noncardiac congenital anatomic abnormality, chromosomal abnormality/syndrome categories with categories 3 to 5 collapsed together, age, and weight) | as a continuous variable (congenital heart surgery): 50/year 100/year 200/year 300/year | OR 2.76 (CrI: 1.43-4.91) OR 1.80 (CrI: 1.04-2.94) OR 1.01 (CrI: 0.55-1.65) OR 0.88 (CrI: 0.60-1.23) | Some concerns |
| LOS=length of stay; eβ=back-transformed coefficients; CI=95% confidence interval; IQR=interquartile range; SD=standard deviation; ECMO=extracorporeal membrane oxygenation; Ref.=reference; E=E-value for unmeasured confounding; SVR=Single Ventricle Reconstruction; TR=tricuspid regurgitation; DHCA=deep hypothermic circulatory arrest; AS/MS/VSD=aortic stenosis, mitral stenosis, ventricular septal defect; CrI=95% credible interval | | | | | |

Table 2. Results of studies examining association between surgeon volume and secondary outcomes

| **Study ID** | **Outcome** | **Analysis/Model  (adjustment)** | **Volume** | **Effect measure** | **Overall risk of bias** |
| --- | --- | --- | --- | --- | --- |
| Anderson 2016 (1) | Median LOS overall | No model | ≤10/year 10-19/year >19/year | 29 (IQR 20-49) 33 (IQR 23-51) 28 (IQR 17-47) p-value=0.267 | Very high risk |
| SVR Trial/ Tabbutt 2012 (5) | Log length of ventilation (days) | Linear regression (gestational age, genetic abnormality, preoperative intubation, left atrial decompression, preoperative shock, TR preoperatively, age, open sternum, operations after Norwood procedure, hospital volume) | ≤5/year 6 to 10/year 11 to 15/year >15/year | 0.33 0.27 0.21 Ref. P=0.008 | High risk |
| SVR Trial/ Tabbutt 2012 (5) | Log time to first extubation (days) | Linear regression (gestational age, left atrial decompression, TR preoperatively, duration of regional cerebral perfusion, ECMO, open sternum, duration of open sternum, operations after Norwood procedure, hospital volume) | ≤5/year 6 to 10/year 11 to 15/year >15/year | 0.54 0.54 0.40 Ref. p<0.001 | High risk |
| SVR Trial/ Tabbutt 2012 (5) | Renal failure | Logistic regression (anomalous pulmonary venous return, preoperative intubation, heart block, open sternum, hospital volume) | ≤5/year 6 to 10/year 11 to 15/year >15/year | OR 0.31 (CI: 0.09-1.09) OR 0.90 (CI: 0.28-2.91) OR 0.20 (CI: 0.06-0.61) Ref. | Some concerns |
| LOS=length of stay; IQR=interquartile range; eβ=back-transformed coefficients; CI=95% confidence interval; SVR=Single Ventricle Reconstruction; Ref.=reference; TR=tricuspid regurgitation; ECMO=extracorporeal membrane oxygenation; | | | | | |

# References

1. Anderson BR, Ciarleglio AJ, Cohen DJ, Lai WW, Neidell M, Hall M, et al. The Norwood operation: Relative effects of surgeon and institutional volumes on outcomes and resource utilization. Cardiol Young. 2016;26(4):683-92.

2. Checchia PA, McCollegan J, Daher N, Kolovos N, Levy F, Markovitz B. The effect of surgical case volume on outcome after the Norwood procedure. J Thorac Cardiovasc Surg. 2005;129(4):754-9.

3. Gong CL, Song AY, Horak R, Friedlich PS, Lakshmanan A, Pruetz JD, et al. Impact of Confounding on Cost, Survival, and Length-of-Stay Outcomes for Neonates with Hypoplastic Left Heart Syndrome Undergoing Stage 1 Palliation Surgery. Pediatr Cardiol. 2020;41(5):996-1011.

4. Schäfer M, McFarland C, Amula V, Truong D, Lambert LM, Griffiths ER, et al. Volume-Outcome Relationship of Norwood Procedures: Insights from the National Pediatric Cardiology-Quality Improvement Collaborative Database. Ann Thorac Surg. 2025;119(5):1045-52.

5. Tabbutt S, Ghanayem N, Ravishankar C, Sleeper LA, Cooper DS, Frank DU, et al. Risk factors for hospital morbidity and mortality after the Norwood procedure: A report from the Pediatric Heart Network Single Ventricle Reconstruction trial. J Thorac Cardiovasc Surg. 2012;144(4):882-95.

6. Welke KF, Karamlou T, O'Brien SM, Dearani JA, Tweddell JS, Kumar SR, et al. Contemporary Relationship Between Hospital Volume and Outcomes in Congenital Heart Surgery. Ann Thorac Surg. 2023;116(6):1233-9.
